# Photoswitchable Cross-Linking in Polymer Gels: Effects on Surface Creasing and Network Relaxation during Swelling

**DOI:** 10.1021/acs.macromol.5c03103

**Published:** 2026-03-26

**Authors:** Alyssa VanZanten, Surbhi Punhani-Schillinger, M. Reed Blocksome, Aditya Ketkar, Shih-Yuan Chen, Michelle M. Driscoll, Robert C. Ferrier, Caroline R. Szczepanski

**Affiliations:** † Department of Chemical Engineering & Materials Science, 3078 Michigan State University, East Lansing, Michigan 48824, United States; ‡ Department of Physics & Astronomy, 3270 Northwestern University, Evanston, Illinois 60208-0001, United States

## Abstract

Polymer gels with photoresponsive cross-links enable tunable mechanics and surface morphologies, making them promising for adaptive materials. While prior work on coumarin cross-linked gels has focused on photomediated events in dilute solution, their network-level mechanical responses remain unclear. Herein, we design PEG hydrogels with both permanent covalent and dynamic coumarin cross-links, allowing *in situ* modulation of cross-linking under wavelength-specific UV light. Real-time FTIR and dynamic mechanical analysis (DMA) show that postcure 365 nm irradiation drives rapid dimerization, increasing storage modulus by up to 69%, whereas cleavage of coumarin cross-links via 254 nm postcure irradiation has a more limited effect due to attenuation in bulk samples. Surface imaging reveals that dynamic cross-linking governs swelling-induced crease formation and evolution. Together, these results establish design principles for hydrogels with programmable mechanics and adaptive surface topographies, enabling light-addressable coatings, mechanically lockable soft actuators, and dynamic biomaterial interfaces.

## Graphical abstract

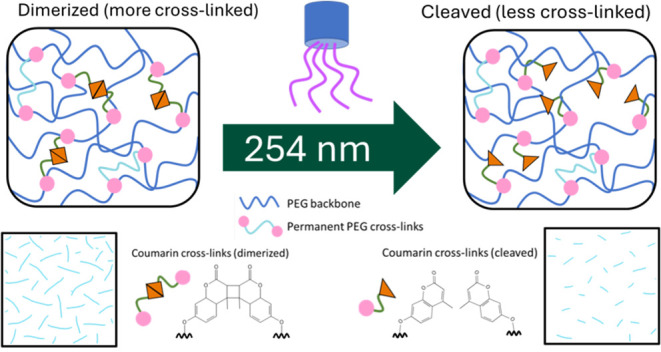

## 1 Introduction

Photoresponsive cross-linking enables *in situ*, spatiotemporal control of network constraints, a valuable strategy for designing smart membranes, fouling-inhibiting coatings, targeted drug-delivery vehicles, and reprocessable plastics. Dynamic covalent bonds that reversibly associate and dissociate under irradiation with light have been integrated into diverse polymer platforms. Such photomediated cross-links impart self-healing and reprocessing,^1–8^ tunable interfacial behavior (e.g., fluorescence, wettability),^9–13^ controlled release,^14–16^ and on-demand actuation.^17–20^ Coumarin moieties dimerize under 365 nm UV light and cleave under 254 nm UV light,^7,21^ offering reversibility and efficient photoluminescence.^1,22^ Recent studies have expanded the use of coumarin photochemistry in surface engineering, additive manufacturing, and biomaterials, including reconstructible gradient hydrogels, visible-light-responsive actuators, and injectable self-healing networks.^5,9,14,18^ These advances demonstrate renewed interest in coumarin-based systems as mechanically active elements rather than purely optical elements, particularly when integrated into bulk networks rather than dilute or supramolecular assemblies.

Gels, soft, three-dimensional cross-linked polymers, provide mobility for reactive groups while maintaining bulk dimensional stability. Cross-linking imposes network constraints that are essential to gel design. Cross-linking results in a three-dimensional matrix that absorbs compatible solvents, but swelling is gradual, generating stresses as solvent diffuses through the network.^23–26^ Property mismatches during solvent diffusion can trigger instabilities such as rupture and surface buckling.^27–40^ Most gels dissipate stresses extensively during swelling,^41–43^ making them ideal for studying stimuli-responsive behavior in which dynamic bond exchange generates internal stresses. Coumarin moieties have been used as gelators or primary cross-linkers in hydrogels,^8,21,22,44–47^ but most studies probe their photodimerization and cleavage in dilute solutions via UV–vis spectroscopy. Dilution removes the topological constraints of a gel network, so such measurements cannot fully capture network-level reaction pathways and kinetics. Additionally, attenuation of UV light in bulk samples can dramatically reduce penetration and efficiency, particularly at 254 nm. This means that it is especially important to consider bulk effects when assessing the coumarin reaction kinetics. Here, we study a hydrogel with both permanent covalent and dynamic coumarin cross-links, which ensure dimensional stability during cross-link cycling. Bulk chemical and physical changes during coumarin dimerization and cleavage are probed by pseudo-real-time Fourier transform infrared (FTIR) spectroscopy and dynamic mechanical analysis (DMA).

Beyond general responsiveness, the ability to reversibly and locally modulate cross-link density in a swollen network is particularly relevant for applications where surface mechanics and stress evolution govern function. Examples include antifouling and flow-regulating coatings, where transient surface creases can alter hydrodynamic slip and contact area;^48,49^ soft microactuators and valves, where light-triggered stiffening enables shape fixation or force amplification;^50^ as well as dynamic cell-instructive biomaterials, where surface topography and stiffness cues regulate adhesion, migration, and differentiation.^51^ In these contexts, materials that decouple bulk integrity from a programmable surface response are especially valuable.

Existing photoswitchable gel systems (most notably those based on spiropyran or azobenzene moieties) typically rely on polarity changes, isomerization-induced swelling, or noncovalent interactions to modulate network properties.^6,10,52,53^ While effective for optical or chemical responsiveness, these systems often suffer from limited mechanical contrast during switching, fatigue under repeated cycling, or creep due to the absence of load-bearing bond formation. In contrast, coumarin dimerization directly introduces covalent cross-links, enabling large, quantifiable increases in elastic modulus (up to 69% in this work) without compromising network integrity.^2,4,7,45,54^

Beyond coumarin chemistry, a variety of strategies have been explored to regulate hydrogel mechanics and surface morphology, including nanocomposite architectures,^55^ multiphase networks,^56^ and alternative photoswitchable motifs.^57^ For example, composite and gradient structures have been used to tailor modulus and swelling-induced patterning in filled or layered soft materials,^55^ while spiropyran- or azobenzene-modified networks leverage polarity changes or isomerization to modulate stiffness and actuation behavior under light.^58,59^ In contrast, the present work uses covalent, photoreversible coumarin cross-links embedded in the backbone of a PEG hydrogel to directly link light-driven bond exchange to the bulk modulus, swelling kinetics, and surface creasing, thereby enabling programmable mechanics and morphology within a single, chemically well-defined network.

Here, we introduce a dual-cross-linked PEG-coumarin hydrogel that integrates permanent poly­(ethylene glycol) diacrylate (PEGDA) cross-links with photoreversible coumarin dimers. This architecture preserves macroscopic dimensional stability while enabling postcure mechanical programming and real-time control of swelling-induced surface instabilities. By coupling bulk FTIR and DMA with *in situ* visualization of surface creasing, this study establishes mechanistic links between light-driven bond exchange, strain relaxation, and evolving surface morphology, providing design principles for adaptive gel-based coatings and soft devices. This study is organized as follows: Section 3.1 presents surface crease images; Section 3.2 compares swelling, photopolymerization, and static mechanics; lastly, Section 3.3 analyzes chemical and mechanical changes during dimerization and cleavage using FTIR and DMA.

## 2 Materials and Methods

### 2.1 Materials

The gel networks investigated (shown in Figure 1) comprised poly­(ethylene glycol) methyl ether acrylate (PEGMA, 400 g/mol, Sigma-Aldrich) as the monomer, poly­(ethylene glycol) diacrylate (PEGDA, 700 g/mol, Sigma-Aldrich) as the permanent cross-linker, and synthesized 7-(2-acryloyloxyethoxy)-4-methylcoumarin (CoumAc) as the photoresponsive secondary cross-linker. 2,2-Dimethoxy-2-phenylacetophenone (DMPA) served as the photoinitiator in all formulations. Commercial monomers (PEGMA and PEGDA) and DMPA were used without further purification. All reagents used for CoumAc synthesis (see below) were purchased from Sigma-Aldrich with the following purity: 4-methylumbelliferone (>98%), potassium carbonate (>99%), 2-bromoethanol (>95%), acryloyl chloride (>97%), triethylamine (>99%), chloroform (>99.8%), *N*,*N*-dimethylformamide (>99.8%), and ethanol (>96%). Deionized (DI) water for swelling experiments was obtained from an in-house source.

**Figure 1:**
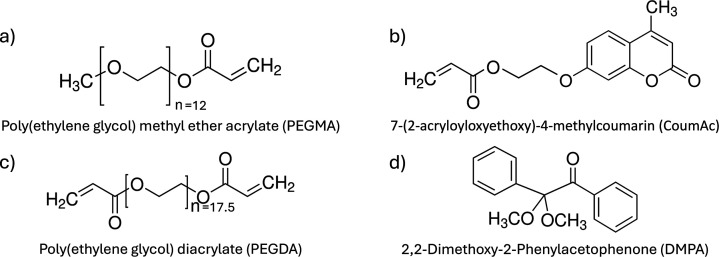
Molecular structure of (a) PEGMA, (b) CoumAc, (c) PEGDA, and (d) DMPA.

### 2.2 Methods

#### 2.2.1 Synthesis of 7-(2-acryloyloxyethoxy)-4-methylcoumarin (CoumAc)

CoumAc synthesis followed Kabb et al. (scheme in Figure S1).^21^ In brief, 4-methyl-umbelliferone (12 g, 68.1 mmol, 1.0 equiv) and potassium carbonate (18.82 g, 136 mmol, 2.0 equiv) were suspended in dimethylformamide under a nitrogen atmosphere, then treated with 2-bromoethanol (7.2 mL, 102 mmol, 1.5 equiv). The mixture was stirred at 90 °C for 18 h, cooled, and combined with ice-cold DI water to form a pink slurry. The pink/off-white precipitate was vacuum filtered, dried, and yielded 7,2-(hydroxyethoxy)-4-methylcoumarin (15 g, 100%, ^1^H NMR in Figure S2).

The intermediate (15 g, 68.1 mmol, 1.0 equiv) was suspended in chloroform under a nitrogen atmosphere, with triethylamine (19.2 mL, 138 mmol, 2.0 equiv) and acryloyl chloride (11 mL, 138 mmol, 2.0 equiv) added. After 1 h of stirring at room temperature, additional triethylamine (9.6 mL, 69.0 mmol, 1.0 equiv) and acryloyl chloride (5.5 mL, 67.7 mmol, 1.0 equiv) were introduced, and the mixture was stirred overnight. The product was washed sequentially with sodium bicarbonate solution (100 mL × 2), DI water (100 mL × 2), and NaCl brine (5 M, 100 mL × 2), dried over sodium sulfate, and gravity filtered. Evaporative recrystallization in ethanol (twice) afforded CoumAc (10 g, 54%, ^1^H NMR in Figure S3, 2-D COSY spectrum in Figures S4 and S5, and Mass-Spec in Figure S6).

#### 2.2.2 Preparation and Curing of PEG:CoumAc Hydrogels

Resin formulations (Table 1) varied the molar fractions of PEGMA, PEGDA, and, in some cases, CoumAc. In the cases where CoumAc was incorporated, the permanent:dynamic ratio was varied as shown in Table 1 to tune cross-link density. The combined mass of these comonomers totaled 99.5 wt % of the resin formulation. All formulations contained 0.5 wt % DMPA to enable free-radical photopolymerization.

**Table 1:** Gel Resin Compositions and Naming Scheme

name	PEGDA mol %	CoumAc mol %	PEGMA mol %
0.5:0.5:99	0.5	0.5	99
0.5:4.5:95	0.5	4.5	95
1:1:98	1	1	98
1:4:95	1	4	95
1:9:90	1	9	90
5:5:90	5	5	90
5:15:80	5	15	80

Components were combined in glass vials, stirred at 60 °C for ∼15 min to homogenize, then injected between glass slides to form 25 mm × 10 mm × 2 mm bars (length × width × thickness). Photopolymerization used either (1) irradiation with 365 nm UV LED (0.1 W/cm^2^, 7 min, ThorLabs Solis LED 365C—this method is similar to that commonly used for photopolymerization of PEG gels, including in our prior work^60^) or (2) 254 nm UV oven (0.0088 W/cm^2^, 9–35 min, Stratagene UV Stratalinker 2400) with samples rotated 180° every minute to ensure a uniform cure. Irradiation continued until Fourier transform infrared (FTIR) spectroscopy confirmed no further decreases in the vinyl peak (6165 cm^–1^). It is important to note that the intensity values listed here are incident and do not reflect the intensity values within the specimens. Attenuation was observed after UV light traveled through the samples, and is reported in Table S1.

#### 2.2.3 Fourier Transform Infrared Spectroscopy

##### 2.2.3.1 Tracking Free-Radical Photopolymerization in Real Time

Real-time Fourier transform infrared (RT-FTIR) spectroscopy (ThermoNicolet, Nicolet iS50) monitored the C=C double bond consumption during photopolymerization. *In situ* measurements used a fiber optic UV source (250–500 nm, 0.1 W/cm^2^, OmniCure series 2000m Excelitas). Pesudo-real-time mode paused 254 nm curing to collect spectra at discrete intervals. Fractional conversion was calculated from the vinyl peak at 6165 cm^–1^ using eq 1.

1 $$f\mathrm{ractional}\;c\mathrm{onversion}=1-\frac{A_{t}}{<A_{0}>}$$

Here, *A*_*t*_ is the peak area at time *t* and <*A*_0_> is the average peak area before irradiation. The rate of photopolymerization (*R*_p_) was calculated as the first derivative of the fractional conversion versus time data, and the maximum rate (*R*_p_^max^) and the time at *R*_p_^max^ were extracted as characteristic descriptions of the photopolymerization reaction.

##### 2.2.3.2 Monitoring CoumAc Dimerization and Cleavage

Pseudo-real-time FTIR in the mid-IR range (400–4000 cm^–1^, resolution of 4 cm^–1^) was used to monitor the chemical changes associated with CoumAc dimerization and cleavage. 0.01 mm thick, free-standing films were formed to mitigate saturation in the mid-IR range. The aromatic C=C double bond at the 3,4-position of the CoumAc moiety has a vibration at approximately 1615 cm^–1^. This signal is reported to appear as a sharp peak in the cleaved state.^9^ Dimerization is reported to reduce the intensity of the C=C double bond peak and form a shoulder at approximately 1575 cm^–1^.^9^ The C=O stretch signal around 1730 cm^–1^, which has a small shoulder at approximately 1775 cm^–1^, is reportedly influenced by the C=C double bond that is present only in the cleaved state.^9^ Therefore, dimerization is expected to shift the C=O peak to higher wavenumbers. Our observation of these chemical changes during the dimerization and cleavage reactions is included in Section 3.3.

#### 2.2.4 Dynamic Mechanical Analysis

Dynamic mechanical analysis (DMA 850, TA Instruments) measured the storage modulus (*E*′), loss modulus (*E*″), and loss factor (tan δ) during temperature sweeps (−60 or −40 to 100 °C, 1 Hz, 0.01% strain, 3 °C/min) in tensile mode. These measurements represent the elastic behavior of the material, the viscous behavior, and the ratio of $\frac{E^{\prime \prime }}{E^{\prime }}$ , respectively. Strain ramp tests measured stress–strain behavior and Young’s modulus (room temperature, 0.2 mm/min) in tensile mode.

#### 2.2.5 Hydrogel Swelling and Morphology Measurements

To quantify the uptake of solvent during gel swelling, mass measurements were collected at various time points. Dry gel mass (*m*_0_) was recorded before immersion in 75 mL of DI water. At set times, gels were removed, blotted, weighed (*m*(*t*)), and returned to the water. Equilibrium swelling mass (*m*_eq_) was reached when the weight stabilized (typically after ∼24 h). Mass swelling ratio (*Q*(*t*)) and normalized swelling ratio ( $\overset{®}{Q\left(t\right)}$ ) were calculated via eqs 2 and 3.

2 $$Q\left(t\right)=\frac{m\left(t\right)}{m_{0}},Q_{0}=\frac{m_{0}}{m_{0}}=1,Q_{\mathrm{eq}}=\frac{m_{\mathrm{eq}}}{m_{0}}$$

3 $$\overset{®}{Q\left(t\right)}=\frac{Q\left(t\right)-Q_{0}}{Q_{\mathrm{eq}}-Q_{0}}=\frac{m\left(t\right)-m_{0}}{m_{\mathrm{eq}}-m_{0}}$$

$\overset{®}{Q\left(t\right)}=0$ at time *t* = 0 because *Q*(*t*) = *Q*_0_. Additionally, $\overset{®}{Q\left(t\right)}=1$ at equilibrium because *Q*(*t*) = *Q*_eq_. Therefore, $\overset{®}{Q\left(t\right)}$ , which increases as swelling progresses, represents the fraction of the total swelling capacity reached after time (*t*) of swelling.

Morphology was imaged *in situ* using an Olympus Microscope ix83 at 2× (6656 μm field) (6656 μm field). Samples were suspended and gently weighted down to prevent movement (Figure S7). During simultaneous UV irradiation and crease imaging, a 254 nm lamp (Transilluminator Handheld UV Lamp BioGlow 254 nm) was placed ∼5 cm from the swelling gel, with an approximate intensity of 0.008 W/cm^2^. Quantitative crease analysis (i.e., measurement of crease segment length) was completed using Python.

## 3 Results and Discussion

The CoumAc-functionalized PEG-based gel network developed here is designed to undergo bulk network transformations upon exposure to UV light after polymerization (Figure 2). Irradiation at 365 nm induces a [2 + 2] cycloaddition, forming cyclobutane-linked CoumAc dimers, whereas 254 nm irradiation cleaves these dimers, restoring CoumAc to its nonpaired state.

**Figure 2:**
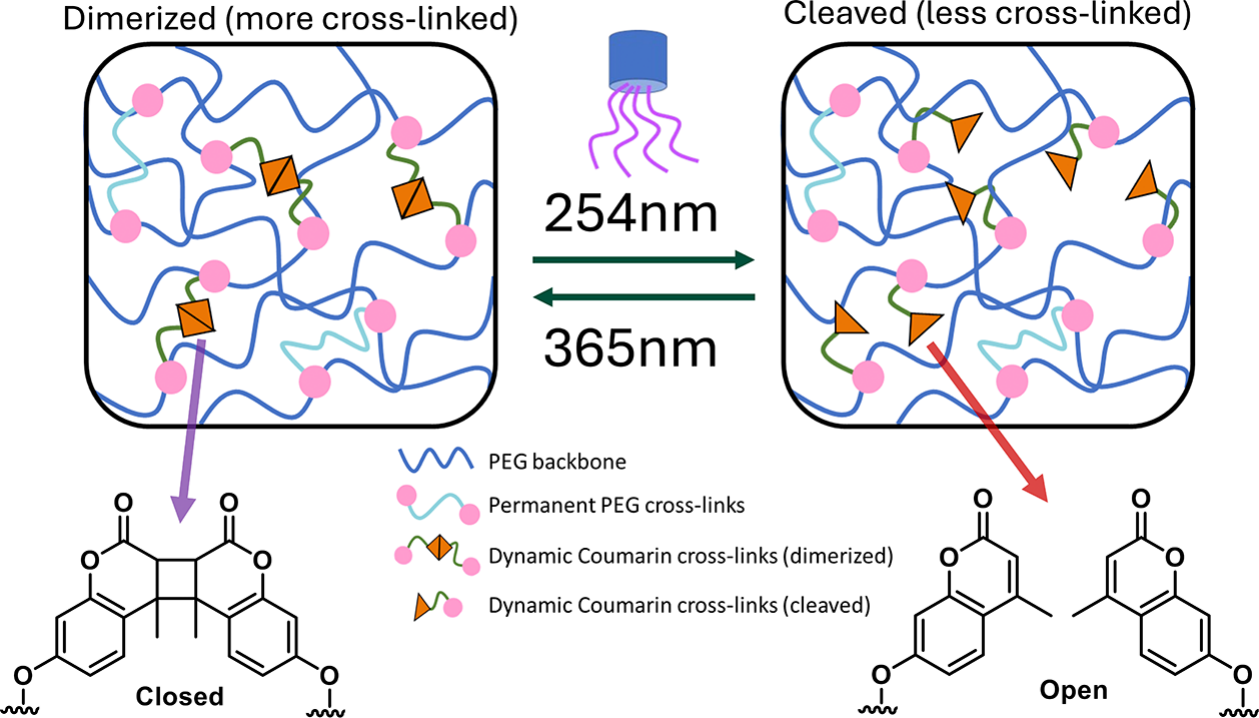
Design of photoresponsive network. Schematic depicting how this dual-cross-linked network is designed to switch between a more constrained state and a less constrained state upon postcure irradiation with UV light.

The base PEG network, covalently cross-linked with PEGDA, maintains structural integrity regardless of postcure irradiation. By incorporation of CoumAc as a secondary, dynamic cross-linker, cross-link density can be reversibly increased via 365 nm dimerization or decreased via 254 nm cleavage. This section outlines (i) how surface instabilities evolve during swelling as a function of CoumAc state (Section 3.1), (ii) baseline kinetic, swelling, and mechanical properties of these networks (Section 3.2), and (iii) chemical/mechanical changes during active photomodulation (Section 3.3). Ultimately, the chemistry demonstrated here offers a route to designing polymer surfaces with precisely programmable topographies via remote, spatiotemporal control. A critical prerequisite for such smart surfaces is a rigorous understanding of how CoumAc incorporation modulates both the bulk mechanical properties and the dynamic network transformations.

### 3.1 Surface Crease Formation during Gel Swelling Based on CoumAc State

Polymer gels often develop surface instabilities such as creasing during swelling.^27,43,60^ To assess how CoumAc cross-linking modulates these instabilities, we performed *in situ* microscopy during swelling in water. Notably, all samples were fully polymerized before swelling, ensuring that the acrylate double bonds were consumed and that subsequent structural evolution could be attributed specifically to the dynamic CoumAc cross-links, rather than acrylate polymerization (representative conversion profiles and polymerization rate profiles are included in the Supporting Information—Figures S8 and S9). Figure 3 presents surface crease patterns for gels photopolymerized and then swollen in water for 320 s (here, swelling proceeds without any UV exposure). Three representative formulations are shown: 1:99 PEG (no CoumAc), 0.5:4.5:95 PEGCoumAc polymerized at 365 nm, and 0.5:4.5:95 PEGCoumAc polymerized at 254 nm. As a reminder, the formulation names indicate the mol % of PEGDA, CoumAc, and PEGMA, respectively. In the micrographs, thick, blurry lines correspond to out-of-focus creases on the top of the sample. Round dark features denote trapped air bubbles beneath the gel. The traced lines highlight in-focus creases on the bottom of the sample. (Unprocessed images are provided in Figure S10.)

**Figure 3:**
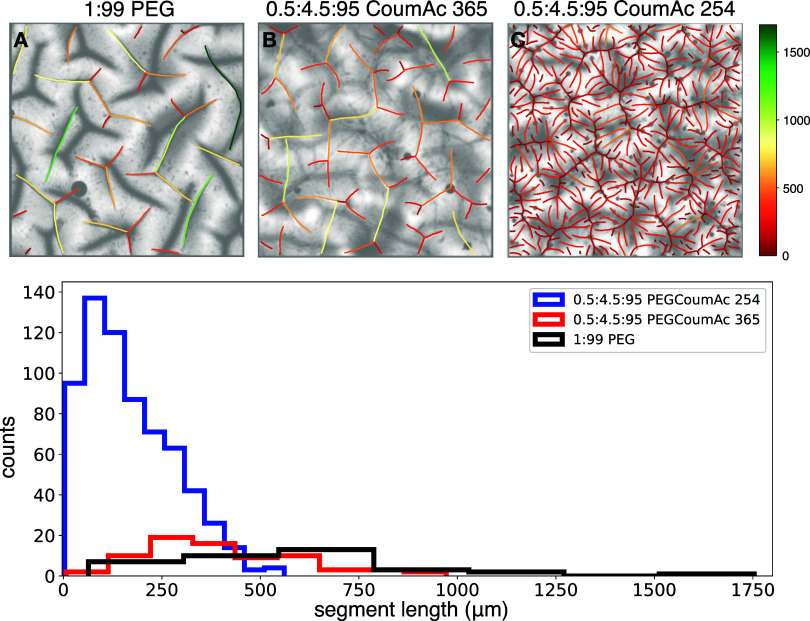
Addition of CoumAc cross-linking, as well as the wavelength of UV light used during photopolymerization, significantly changes crease patterns. Microscope images measuring 3.328 mm × 3.328 mm compare the surface crease patterns on the surface of previously polymerized gels after 320 s of swelling in water for the (A) 1:99 PEG formulation, as well as the 0.5:4.5:95 PEGCoumAc formulation (B) polymerized at 365 nm and (C) polymerized at 254 nm. Crease tracings are color-coded based on segment length (see scale, right), with the associated distribution of segment lengths for each sample plotted below. The high density of short creases is reflected in the blue series for the 0.5:4.5:95 formulation cured with 254 nm irradiation. Original microscope images are included in Figure S10.

Creases arise when the gel surface buckles under compressive stresses, leading to regions where the surface folds and contacts itself. These instabilities originate from gradients in swelling: as solvent is absorbed, the highly swollen outer layer is constrained by the initially unswollen core, producing depthwise stress gradients. Comparing the 1:99 PEG sample (Figure 3A) with the 0.5:4.5:95 PEGCoumAc sample polymerized at 365 nm (Figure 3B), we observe that the presence of CoumAc modestly increases the density of surface creases, although the general branched morphology—points where three creases meet—is preserved. This similarity in morphology is supported by the segment length analysis (bottom panel, Figure 3). Specifically, a slightly higher density of short creases is observed in the 0.5:4.5:95 PEGCoumAc sample (red series) compared with the PEG control (black series). Notably, the crease density increase occurs despite similar swelling ratios at 300 s (*Q*_*t*_ of 1.95 for 1:99 PEG, 1.71 for 0.5:4.5:95 365poly, and 1.68 for 0.5:4.5:95 254poly—see Figure S11). This indicates that differences in creasing morphology cannot be attributed solely to differences in swelling kinetics but rather to changes in the network structure induced by the CoumAc cross-link and polymerization conditions. In contrast, the same CoumAc formulation polymerized at 254 nm exhibited a markedly different brush-like pattern with very high crease density (Figure 3C, blue series in crease segment analysis)—implying greater compressive stresses, consistent with classical wrinkling theory linking stress magnitude to feature wavelength and density.^60^ This result is surprising given that gels polymerized at 254 nm do not contain CoumAc dimers. These differences underscore the profound influence of photopolymerization conditions and dynamic cross-linking on the evolution of surface instabilities. The pronounced wavelength- and history-dependent differences in fold density and morphology suggest a route to light-addressable surface patterning, relevant for switchable adhesion, wetting, or friction-control coatings.

The brush-like, high-density creases observed in the 0.5:4.5:95 PEGCoumAc gel cured at 254 nm (Figure 3C) likely arise from a combination of increased network homogeneity and enhanced near-surface cross-link density. Slower, attenuated 254 nm curing allows PEG chains and CoumAc groups to explore conformational space longer before gelation, promoting a more uniform, tightly cross-linked network, as reflected in the higher storage moduli of 254 nm cured gels compared with their 365 nm counterparts (Table S2). During swelling, this more homogeneous but stiffer network can sustain larger compressive stresses near the surface, which, together with possible gradients in cross-link density induced by the attenuated 254 nm light, yield shorter, more closely spaced creases. This is consistent with classical wrinkling theories linking higher compressive stress and stiffness to a reduced wrinkle wavelength and increased feature density. Among several potential explanations (e.g., purely kinetic trapping vs cross-link gradients), this stiffness- and heterogeneity-based mechanism best matches the observed modulus, swelling, and morphological trends.

### 3.2 Network Formation Kinetics and Swelling Dynamics

To assess how CoumAc cross-linking influences swelling-induced instabilities, we first established baseline chemical and mechanical properties directly following free-radical polymerization. Real-time Fourier transform infrared (RT-FTIR) spectroscopy was used to quantify the effect of the CoumAc content and irradiation wavelength during photopolymerization on network formation kinetics. Table 2 reports the maximum polymerization rate (*R*_p_^max^) for each formulation cured at either 365 or 254 nm, along with the corresponding time to *R*_p_^max^ and final fractional conversion. A representative kinetic trace is provided in Figure S8.

**Table 2:** Rate of the Free-Radical Polymerization Reaction Is Significantly Faster at 365 nm Than at 254 nm^a^

PEGDA (mol %)	CoumAc (mol %)	*R*_p_^max^ (1/min)	Time at *R*_p_^max^ (min)	Acrylate fractional conversion at *R*_p_^max^	Final fractional conversion
		Photopolymerized at 365 nm			
0.5	0.5	1.24 ± 0.47	0.33 ± 0.21	0.22 ± 0.10	1
0.5	4.5	1.93 ± 0.10	0.16 ± 0.018	0.24 ± 0.019	1
1	0	4.44 ± 0.06	0.11 ± 0.005	0.25 ± 0.001	1
1	1	2.57 ± 1.29	0.16 ± 0.08	0.16 ± 0.08	1
1	4	1.85 ± 0.55	0.15 ± 0.02	0.22 ± 0.04	1
1	9	1.05 ± 0.1	0.30 ± 0.12	0.21 ± 0.08	1
5	0	4.87 ± 0.06	0.12 ± 0.004	0.29 ± 0.004	1
5	5	2.80 ± 1.31	0.11 ± 0.07	0.20 ± 0.11	1
5	15	0.85 ± 0.05	0.53 ± 0.01	0.33 ± 0.01	1
10	0	5.19 ± 0.64	0.14 ± 0.009	0.33 ± 0.014	1
		Photopolymerized at 254 nm			
0.5	0.5	0.60 ± 0.13	0.72 ± 0.64	0.14 ± 0.09	0.97 ± 0.04
0.5	4.5	0.36 ± 0.22	1.96 ± 0.58	0.23 ± 0.03	0.96 ± 0.03
1	1	0.44 ± 0.04	1.51 ± 0.42	0.28 ± 0.12	0.98 ± 0.0003
1	4	0.24 ± 0.16	5.05 ± 4.27	0.40 ± 0.02	0.98 ± 0.01
1	9	0.63 ± 0.88	4.70 ± 2.36	0.29 ± 0.14	0.99 ± 0.03
5	5	0.28 ± 0.08	3.72 ± 0.97	0.41 ± 0.01	0.99 ± 0.01
5	15	0.083 ± 0.03	7.75 ± 2.91	0.31 ± 0.07	0.99 ± 0.01

a Photopolymerization kinetics parameters obtained *via* RT-FTIR for all PEGCoumAc formulations for curing at both 365 and 254 nm as well as for the base PEG system containing no CoumAc.

Formulations containing CoumAc exhibit slower polymerization kinetics than the base PEG system, as evidenced by markedly higher *R*_p_^max^ values for CoumAc-free formulations (see Table 2). Increasing the CoumAc fraction progressively reduces *R*_p_^max^ and increases the time required to reach this maximum rate. This trend likely arises from the steric bulk of the CoumAc moiety, which can hinder favorable molecule orientation for rapid photopolymerization, particularly once the forming PEG network restricts segmental mobility.

Polymerization kinetics differ substantially between the two cure wavelengths due to the optical properties of the photoinitiator DMPA, which absorbs roughly 10-fold more strongly at 254 nm than at 365 nm.^61^ While higher absorbance generally accelerates radical generation, the effective rate is also governed by light intensity. Here, the 365 nm source delivered 0.1 W/cm^2^—over ten times the intensity of the 254 nm source (0.0088 W/cm^2^). Furthermore, the penetration depth of UV irradiation scales with wavelength (e.g., shorter wavelengths correspond to shorter penetration depths).^62^ The data summarized in Table 2 were collected on samples with thicknesses of ∼2 mm, which were chosen to enable capture and analysis of swelling-induced instabilities (Figure 3). Overall, these combined factors of intensity, absorption, and penetration depth resulted in faster curing at 365 nm despite DMPA’s greater absorbance at 254 nm. Accordingly, *R*_p_^max^ values at 365 nm were approximately an order of magnitude greater than those at 254 nm (e.g., for the 0.5:0.5:99 formulation, *R*_p_^max^ = 1.24 at 365 nm, but *R*_p_^max^ = 0.6 at 254 nm). This trend was consistent across all of the formulations. Importantly, all samples achieved ≥0.96 final fractional conversion regardless of wavelength, indicating near-complete acrylate consumption. Nonetheless, the slower network formation at 254 nm suggests significant differences in final network topology compared with the 365 nm cured gels.

The fractional conversion at *R*_p_^max^ reflects the state of the network when autodeceleration begins (see Figure S9 for representative *R*_p_ vs fractional conversion plots). Beyond *R*_p_^max^, chain mobility becomes increasingly restricted by rising viscosity and diffusion limitations imposed by the forming network.^63,64^ For 365 nm cured samples, fractional conversions at *R*_p_^max^ were similar across formulations, whereas 254 nm cured samples displayed greater variation, consistent with the broader kinetic differences observed. Notably, at a higher overall cross-link fraction, *R*_p_^max^ is shifted to higher degrees of conversion. Variations in the viscosity environment as a function of the CoumAc fraction may also support the shift in *R*_p_^max^ to higher degrees of conversion. Modest increases in viscosity can enhance autoacceleration (e.g., diffusion-limited termination).

To contextualize these kinetic trends, the rate of initiation (*R*_i_) was estimated using eq 4, which incorporates the initiation efficiency (*f*), quantum yield (*Q*), molar absorptivity (ϵ), concentration of initiator ([*I*]), light intensity (*I*_0_), and wavelength (λ)^65^

4 $$R_{i}=\frac{2\mathrm{fQ}\epsilon \left[I\right]I_{0}\lambda }{0.1196\left(J\cdot m/\mathrm{mol}\right)}$$

Using reported molar absorptivities for DMPA,^66^ an initial efficiency *f* = 1 (valid at early reaction stages^62^), and a quantum yield *Q* = 0.42,^67^ the ratio of *R*_i_ at 365 nm versus 254 nm irradiation is calculated by eq 5

5 $$\frac{R_{i,365}}{R_{i,254}}=\frac{\epsilon_{365}I_{0,365}\lambda_{365}}{\epsilon_{254}I_{0,254}\lambda_{254}}=\frac{(110)\left(0.1\frac{W}{cm^{2}}\right)\left(365\;\mathrm{nm}\right)}{(8830)\left(0.0088\frac{W}{cm^{2}}\right)\left(254\;\mathrm{nm}\right)}=0.20$$

This calculation yields $\frac{R_{i,365}}{R_{i,254}}=0.2$ , illustrating that DMPA’s higher absorbance at 254 nm favors more rapid polymerizations, provided irradiation is not significantly attenuated within the sample. As a result, these different irradiation and curing conditions are expected to produce networks with distinct microstructures^68^ and potential gradients in network heterogeneity.

Equation 4 assumes a thin-film geometry with limited reduction in UV intensity through the sample depth. However, our study focuses on bulk specimens with thicknesses of 2 mm, where the attenuation of irradiation is significant. This attenuation is demonstrated by the intensity drop measurements provided in Table S1. Thus, the calculated *R*_i_ values cannot be correlated with our kinetic analyses (Table 2). However, the ratio $\frac{R_{i,365}}{R_{i,254}}$ offers useful insight into how irradiation source characteristics influence network kinetics; it does not fully account for the reduction in effective initiation rate in thicker samples due to light attenuation.

To probe how these differing kinetic environments impact bulk measurements such as swelling behavior, we measured sample mass at discrete time points during immersion in water and calculated the normalized swelling ratio $\overset{®}{Q\left(t\right)}$ (eq 3). Figure 4 compares early stage swelling (first 200 min) for formulations with identical total cross-linker content but varying PEGDA:CoumAc ratios and cure wavelengths.

**Figure 4:**
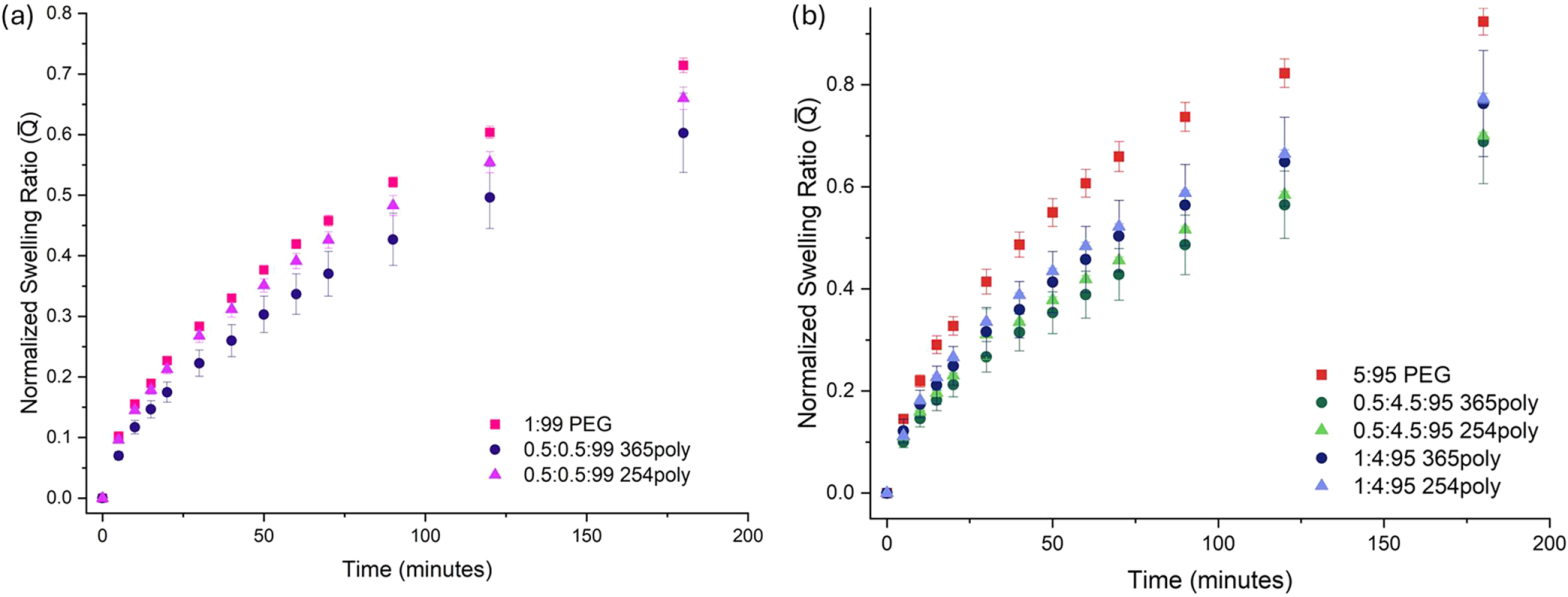
Swelling behavior of PEGCoumAc samples polymerized at either 365 or 254 nm is compared with the base PEG system. (a) The normalized swelling ratio ( $\overset{®}{Q\left(t\right)}$ ) for PEGCoumAc formulations containing 0.5 mol % CoumAc is compared with the 1:99 PEG formulation (polymerized at 365 nm). (b) The normalized swelling ratio ( $\overset{®}{Q\left(t\right)}$ ) of PEGCoumAc formulations containing 0.5 and 1 mol % CoumAc is compared with the 5:95 PEG formulation (polymerized at 365 nm).

For a total cross-linker content of 1 mol % (Figure 4a), replacing a portion of PEGDA with CoumAc reduced $\overset{®}{Q\left(t\right)}$ relative to the PEGDA-only control (1:99 PEG) during early swelling, indicating a slower approach to equilibrium. At a higher cross-linker fraction (5 mol %, Figure 4b), a small increase in CoumAc fraction measurably decreased $\overset{®}{Q\left(t\right)}$ . Furthermore, Figure 5 shows that going from 4 mol % CoumAc to 4.5 mol % CoumAc increases the swelling ratio at equilibrium. This increase in swelling capacity is likely due to the decrease in PEGDA fraction and indicates that the reduction in $\overset{®}{Q\left(t\right)}$ corresponds with a longer time required to reach a higher equilibrium swelling ratio. Across all cases, 254 nm cured samples showed marginally higher $\overset{®}{Q\left(t\right)}$ in the first 200 min of swelling, though not always significant. At equilibrium, swelling ratios *Q*_eq_ (Figure 5) were higher for all CoumAc-containing gels than for PEGDA-only analogues of equal cross-link density, again reflecting CoumAc’s lower cross-linking efficiency.

**Figure 5:**
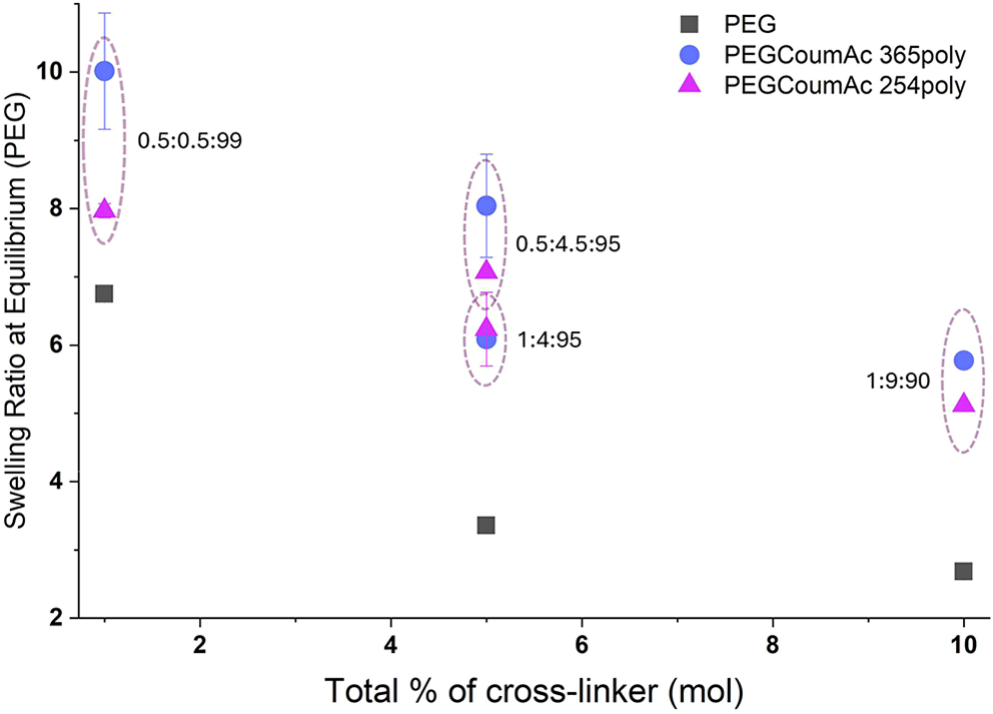
Swelling ratio at equilibrium (*Q*_eq_) is compared between PEG-only formulations (polymerized at 365 nm) and PEGCoumAc formulations with equivalent total mol % of cross-linker (polymerized at either 254 or 365 nm).

Interestingly, 254 nm cured gels absorbed less water at equilibrium than 365 nm cured counterparts, opposite to the expected trend if 365 nm curing produced more CoumAc dimers. This reversal suggests that the cure wavelength affects network topology in ways not captured by a simple cross-link count. Collectively, these results establish direct links between photopolymerization conditions, curing kinetics, and the resulting swelling properties of PEGCoumAc gels, providing the baseline context for interpreting photoresponsive changes that can be induced via *postcure* irradiation.

### 3.3 Photoresponsive Cross-Linking Kinetics: Chemical and Mechanical Changes

Initial, static mechanical properties, measured via DMA at 26 °C (reported in Figure 6 at postcure irradiation time equal to zero), revealed higher storage moduli for gels polymerized via 254 nm irradiation than for analogous counterparts polymerized at 365 nm, contrary to expectations that dimerization during 365 nm curing would stiffen the network. This paradox can be rationalized by differences in polymerization kinetics. The slower network growth at 254 nm allows polymer chains and CoumAc moieties more time to explore conformational space before gelation, promoting a more homogeneous cross-link distribution. Such structural uniformity enhances load-bearing efficiency, producing higher macroscopic stiffness. By contrast, rapid curing at 365 nm may kinetically trap a less ordered, more heterogeneous network, lowering storage modulus despite higher potential cross-link density from CoumAc dimerization.

**Figure 6:**
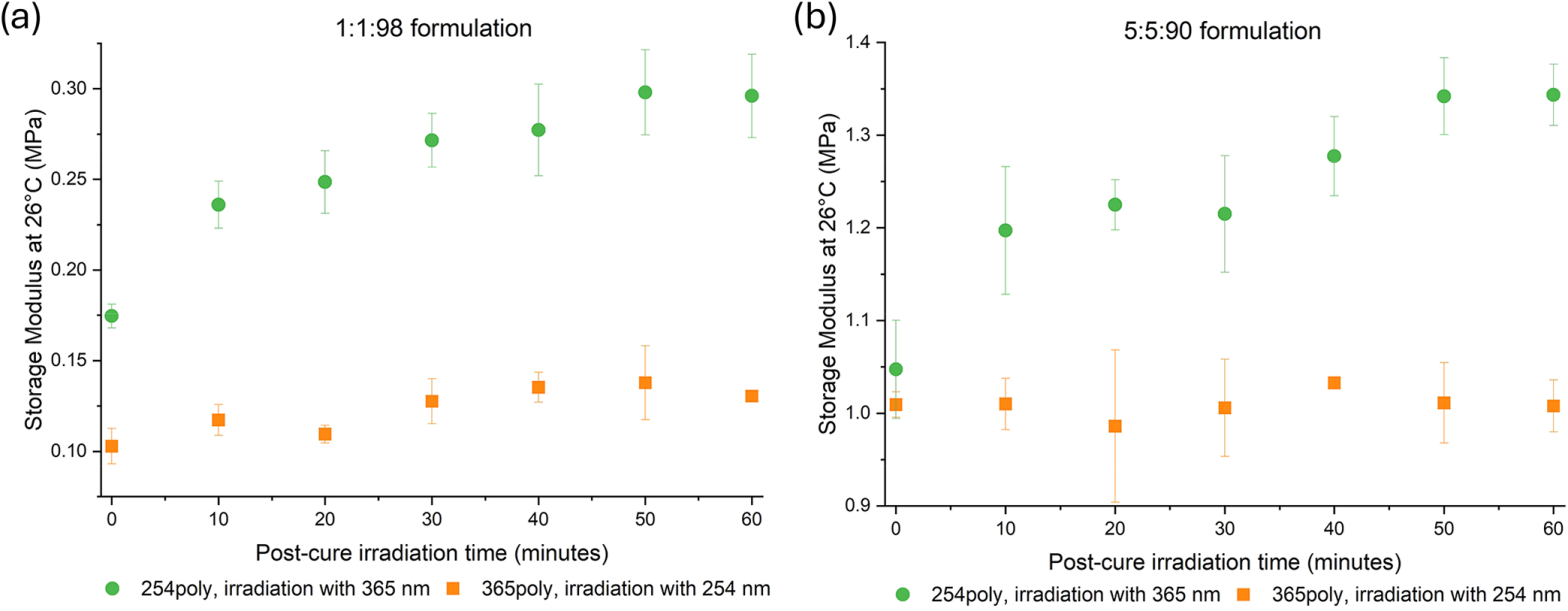
Postcure irradiation with 365 nm significantly increases storage modulus at room temperature, but no change is observed during irradiation with 254 nm. The storage modulus at 26 °C is measured at 10 min intervals during postcure irradiation for (a) 1:1:98 samples and (b) 5:5:90 samples. Samples polymerized at 365 nm are subsequently irradiated with 254 nm light (orange circle) to induce cleavage, while samples polymerized at 254 nm are irradiated with 365 nm light (green squares) to induce dimerization.

Postcure irradiation triggering either CoumAc dimerization or cleavage is anticipated to alter both the molecular-scale and the bulk mechanical properties as a function of the irradiation time. To quantify macroscopic effects, we monitored storage modulus (*E*′) at 26 °C via DMA during postcure UV exposure. Figure 6 compares two reciprocal experiments: (1) cleavage: 254 nm irradiation of gels polymerized at 365 nm (orange series, squares) and (2) dimerization: 365 nm irradiation of gels polymerized at 254 nm (green series, circles). Data are shown for 1:1:98 gels (Figure 6a) and 5:5:90 gels (Figure 6b). In the cleavage case, storage modulus is essentially unchanged, implying that 7 min of 365 nm curing did not generate a substantial fraction of CoumAc dimers. Thus, subsequent irradiation with 254 nm had minimal impact on bulk stiffness. The observed attenuation of 254 nm UV light through the sample depth likely also limited any coumarin cleavage to the surface of the sample.

In contrast, postcure dimerization via 365 nm irradiation produced substantial stiffening. After 60 min, *E*′ increased by 69% in the 1:1:98 formulation and by 28% in the 5:5:90 formulation. Strikingly, ∼50% of this total increase occurred within the first 10 min (35 and 14%, respectively), highlighting the rapid onset of network reinforcement once dimerization begins. These results indicate that the CoumAc moieties embedded within the network retain sufficient mobility to have reactive encounters (i.e., [2 + 2] cycloadditions) that alter the bulk network properties. Even a modest loading (1 mol % CoumAc) can induce a pronounced macroscopic response, with *E*′ rising by ≥65%, demonstrating that low concentrations of dynamic cross-linker can effect large changes in bulk stiffness.

To confirm that these macroscopic properties (i.e., stiffening) are correlated with variations in chemical structure (i.e., dimerization), we used pseudo-real-time mid-IR FTIR on 5:5:90 PEGCoumAc films of ∼0.01 mm thickness (to minimize absorbance saturation). In all experiments, a single sample was evaluated to minimize differences in functional group concentrations as a function of variations in sample preparation. This analysis provides confirmation of the cleavage and dimerization events; however, given the difference in specimen geometry between the samples assessed via mid-IR spectroscopy and those characterized for macroscopic stiffness, the absolute changes in peak intensity/area cannot be compared directly (Figure 6). Two diagnostic features of the coumarin chromophore were tracked: (1) the aryl conjugated carbon–carbon double bond stretch (≈1616 cm^–1^) and (2) the carbonyl (C=O) stretch (≈1740 cm^–1^).^9^ Upon dimerization, the carbon-carbon double bond signal is expected to diminish and shift slightly upward in wavenumber, while the carbonyl peak shifts to higher wavenumbers due to altered conjugation.

As shown in Figure 7a, the carbon–carbon double bond peak centered at 1616 cm^–1^ decreased markedly in intensity and shifted to ≈1624 cm^–1^ during 365 nm irradiation. Concurrently, the C=O peak shifted modestly toward higher wavenumbers (Figure 7b). Most spectral evolution occurred within the first 60 min, with negligible change thereafter. This is overall consistent with the stiffening kinetics observed by DMA (Figure 6).

**Figure 7:**
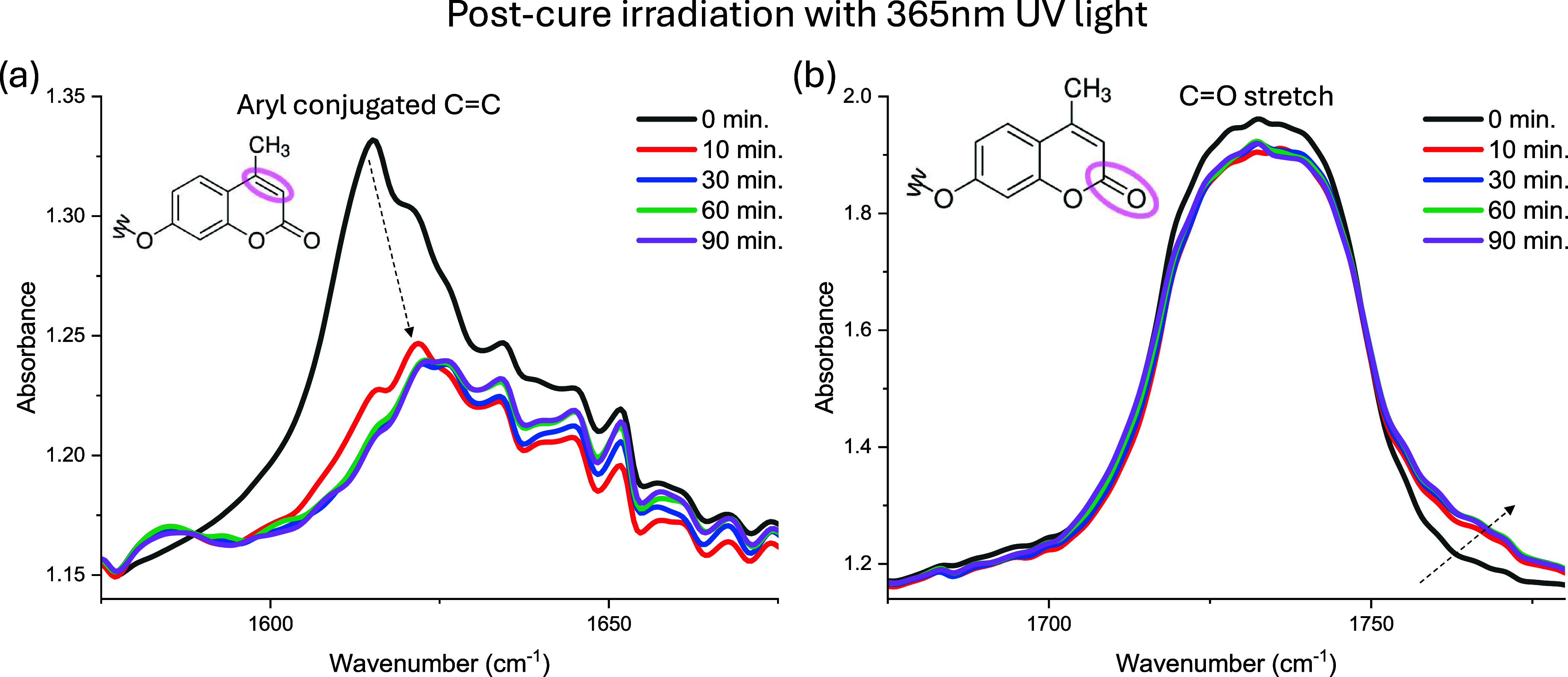
5:5:90 PEGCoumAc film (0.01 mm thick) polymerized with 254 nm UV light for 2 min underwent subsequent 90 min of postcure irradiation with 365 nm to induce dimerization of CoumAc molecules. (a) The peak corresponding to the carbon-carbon double bond present in the nonbonded CoumAc molecule is highlighted and (b) features the peak corresponding to the C=O stretching signal.

For cleavage kinetics, films were polymerized at 365 nm (90 min) to maximize dimer content, then exposed to 254 nm irradiation. Initially, the 1616 cm^–1^ carbon-carbon double bond peak decreased, but by 60 min, a new shoulder emerged between 1637 and 1660 cm^–1^ (see Figure 8a), likely reflecting vibrational modes of carbon centers adjacent to the carbonyl in the cleaved state—supporting dimer scission. In parallel, the C=O band broadened (Figure 8b), which is also consistent with structural reorganization.

**Figure 8:**
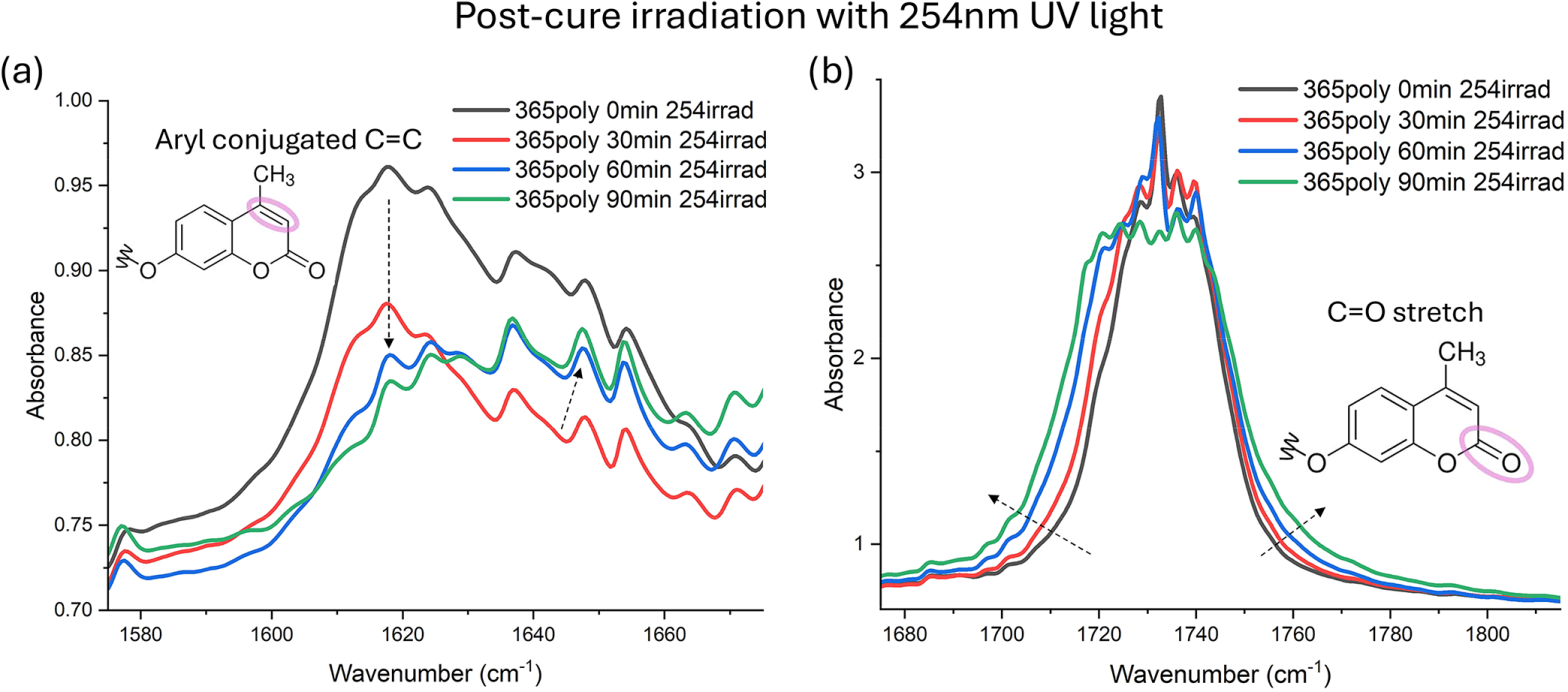
5:5:90 PEGCoumAc film (0.01 mm thick) polymerized with 365 nm UV light for 90 min underwent subsequent 90 min of postcure irradiation with 254 nm to induce cleavage of CoumAc molecules. (a) The peak corresponding to the C–C double bond present in the nonbonded CoumAc molecule is highlighted and (b) features the peak corresponding to the C=O stretching signal.

FTIR data indicate that both the dimerization and the cleavage in the bulk network require ≥60 min to approach steady state. The unexpectedly slow cleavage is attributed to low 254 nm intensity and limited penetration depth into the millimeter-scale network (intensity measurements are provided in Table S1). Previous investigations by Kabb et al. into cleavage kinetics via UV–vis report that 5–15 min of 254 nm irradiation is required to effect a significant increase of the absorbance of the carbon-carbon double bond reformation.^21^ By incorporating CoumAc into this PEG gel network, topological constraints are effectively increased, meaning that we would expect cleavage in our system to be slower than that reported via UV–vis analysis. Nonetheless, 10 min of 365 nm exposure suffices to drive substantial dimerization for CoumAc moieties already in close proximity (Figure 6); more distant ones require longer times, with overall rates depending on total cross-link density and CoumAc loading.

To assess reversibility, 1:9:90 bars (25 mm × 10 mm × 2 mm) polymerized at 365 nm for 77 min were subjected to three consecutive cleavage/dimerization cycles consisting of (i) irradiation at 254 nm for 90 minutes and (ii) irradiation at 365 nm for 90 minutes. Young’s modulus was measured after each step to track bulk property evolution (Figure 9).

**Figure 9:**
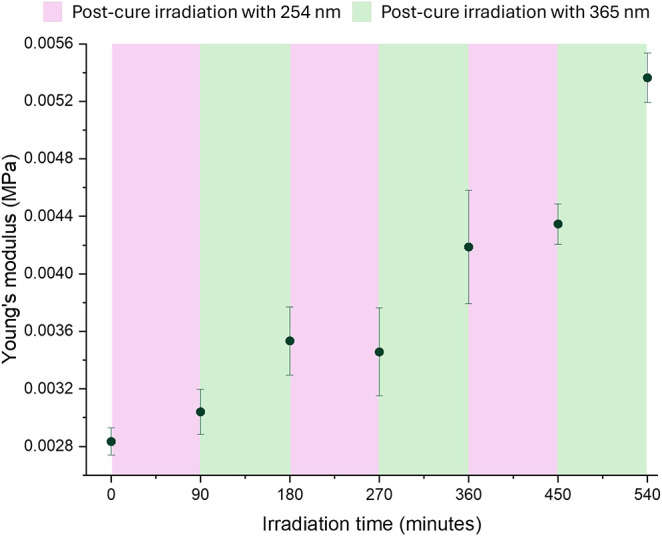
Cycling between 254 and 365 nm irradiation revealed that cleavage has no significant impact on bulk mechanical properties. A 1:9:90 sample polymerized at 365 nm for 77 min was irradiated, postcured with 254 nm UV light for 90 min (pink regions), and then postcured with 365 nm for 90 min (green regions). This procedure was repeated for 3 total cycles.

With this experiment, 254 nm irradiation steps had a negligible effect on Young’s modulus, despite the expectation of modulus loss from dimer cleavage. Combined with FTIR evidence (Figure 8), this suggests that cleavage at 254 nm is largely confined to the surface, given the attenuation through the sample depth, meaning that insufficient photons are delivered to measurably alter bulk load-bearing structure. Conversely, each 365 nm irradiation step increased modulus further. After 270 min cumulative exposure, modulus had doubled relative to postcure baseline, implying that many CoumAc groups remained available for dimerization well after initial cure. Irradiation at 365 nm on the scale of tens of hours would likely be required to reach full dimerization in the network. The slower bulk kinetics compared with thin-film FTIR curves (Figure 7) highlight that network-wide dimerization is diffusion limited in thick specimens. Overall, these results show that while efficient bulk stiffening via dimerization is achievable on hour-scale time scales, bulk softening via cleavage is minimal under current 254 nm conditions. This limited softening is further supported when estimating the attenuation of 254 nm light through a 2 mm thick specimen using open-source simulation tools.^69^ As highlighted in Figure S12, 254 nm irradiation has significantly limited attenuation (i.e., 90% decrease in intensity at a depth of ∼25 μm). Despite the limited softening response, the rapid subhour stiffening achieved with low coumarin loadings is particularly advantageous for on-demand mechanical locking in soft actuators or shape-morphing elements, where fast reinforcement without additional chemical inputs is required.

It is important to distinguish which conclusions are robust to specimen thickness and which are thickness-sensitive. The FTIR film measurements (thickness ∼0.01 mm) confirm that coumarin dimerization under 365 nm and cleavage under 254 nm both proceed as expected, with characteristic changes in the aryl C=C and carbonyl bands (Figures 7 and 8). By contrast, the extent and spatial uniformity of photomediated events in 2 mm thick gels are strongly thickness-sensitive due to the attenuation and diffusion-limited network reorganization. Bulk stiffening via dimerization requires tens of minutes to hours (Figure 6), and cleavage under 254 nm produces detectable spectral changes (Figure 8) but negligible changes in the macroscopic modulus (Figure 9), consistent with a thin active layer surface (Figure S12). Thus, trends in modulus increase and in swelling-induced creasing with increasing dimer content are robust, whereas absolute conversion and the depth profile of dimerization/cleavage depend sensitively on sample thickness and irradiation protocol.

Under our current conditions, the 254 nm protocol is effectively surface-dominated. Intensity measurements and attenuation simulations indicate that 254 nm light loses approximately 90% of its incident intensity within the top ∼25 μm of a 2 mm specimen (Figure S12 and Table S1), such that photon delivery to the bulk is strongly limited. Combining this attenuation with the negligible changes in bulk storage and Young’s moduli during 254 nm exposure (Figures 6 and 9) suggests that the “active layer” for coumarin cleavage is confined to a tens-of-microns-thick surface region, while the underlying network remains largely unchanged. This surface-localized cleavage rationalizes the coexistence of minimal bulk softening with pronounced changes in the surface creasing response under 254 nm protocols, as highlighted below.

To probe the impact of polymerization time and postcure UV exposure on crease evolution, *in situ* microscope images were collected during swelling for varying 5:5:90 PEGCoumAc samples polymerized at 365 nm (Figure 10). We first compare crease evolution during swelling between a sample photopolymerized at 365 nm for a short irradiation period (7 min, Figure 10a–c) and a longer irradiation period (77 min, Figure 10d–f). These two curing protocols were informed by the kinetic data in Figure S8 and the storage modulus data in Figure 6. Our kinetic profiles highlight that a 7 min curing period is sufficient for complete double bond consumption (i.e., 1.0 fractional conversion of acrylate functionalities), while our DMA data informs that the storage modulus plateaued after 70 min of additional 365 nm irradiation. Comparing these two sets of images reveals qualitative similarities between the transient crease evolution: dense crease formation at early time points (first few seconds of swelling) as well as similar morphologies (i.e., lack of branching) over the following minutes. This comparison also reveals that prolonged 365 nm exposure time for photopolymerization (77 min), which increases dimer content, results in creases that are both longer and more densely packed at 185 s of swelling. This suggests that a higher dimer content suppresses swelling kinetics and maintains elevated compressive stresses for a longer duration during swelling.

**Figure 10:**
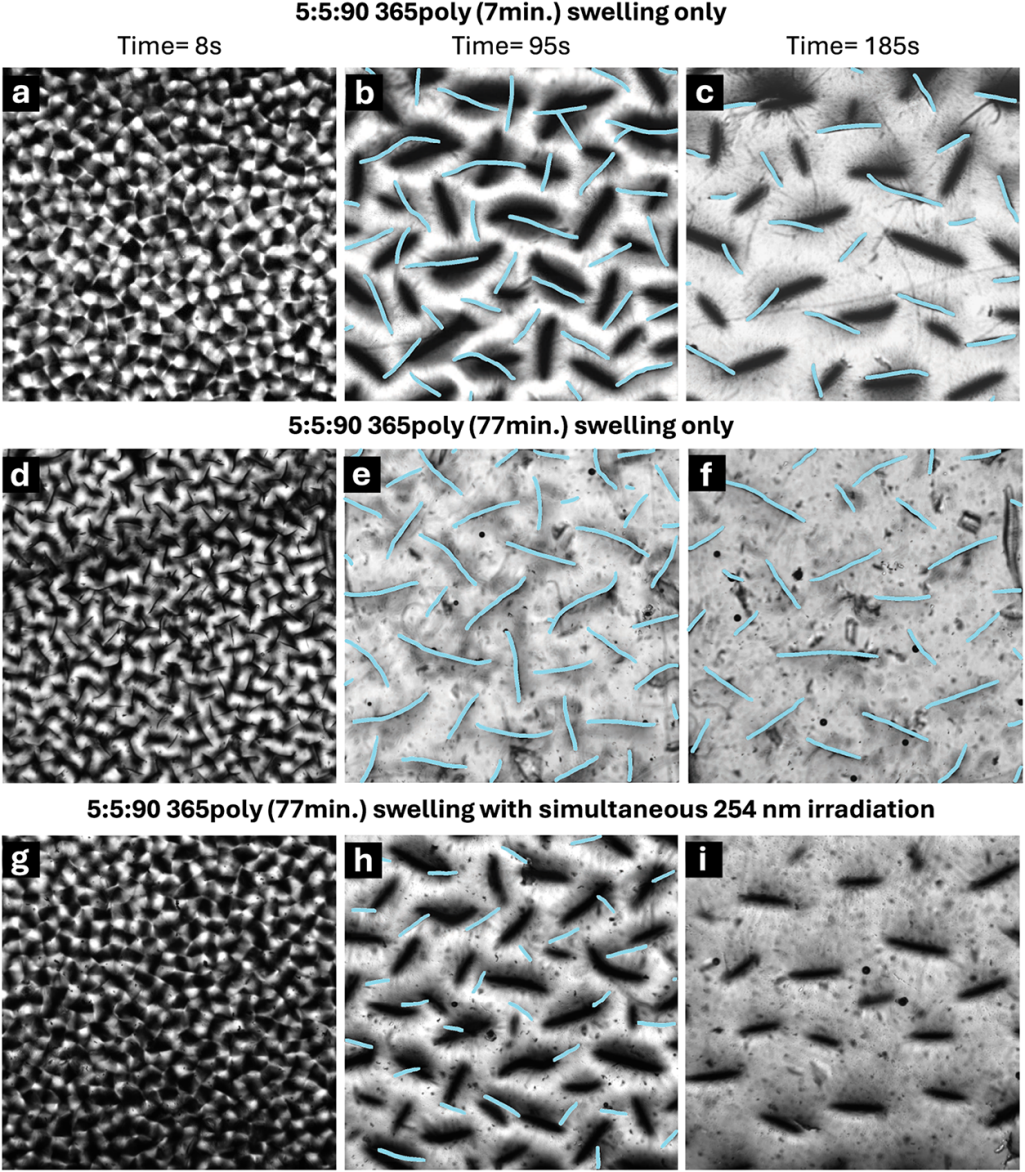
*In situ* microscopy reveals that irradiation with 254 nm UV light during swelling increases the rate of crease evolution. Three 5:5:90 PEGCoumAc samples with varying polymerization conditions and postcure exposure are shown at (a, d, g) 8, (b, e, h) 95, and (c, f, i) 185 s of swelling. (a–c) A sample polymerized at 365 nm for 7 min, (d–f) a sample polymerized at 365 nm for 77 min, and (g–i) the impact of simultaneous 254 nm irradiation during swelling for a sample polymerized at 365 nm for 77 min. All images show a field of view of 3.328 mm × 3.328 mm.

Given the mid-FTIR results for thinner specimens of this formulation (Figure 8), we hypothesize that at a minimum, photomediated bond-exchange events will arise at the surface of our gel samples, which may alter dynamic crease evolution during swelling if a sample is exposed to 254 nm irradiation *during* swelling (Figure 10g–i). Our imaging data supports this conclusion: a sample cured under365 nm irradiation for 77 minutes (producing a high dimer content) undergoes rapid surface-crease disappearance upon exposure to 254 nm light during swelling, as CoumAc cleavage is activated and all in-focus creases vanish by 185 s (Figure 10i). This observation indicates that dimer cleavage by 254 nm light accelerates swelling and relieves surface compressive stresses during the initial swelling period.

While we attribute the rapid disappearance of creases primarily to coumarin dimer cleavage at the surface, 254 nm irradiation can in principle induce additional UV-driven changes (e.g., photooxidation or minor chain scission) that might also help alter near-surface mechanics. Several observations help bound these alternative contributions: (i) 254 nm exposure of 365 nm cured bulk specimens produced negligible changes in bulk modulus over similar time scales (Figure 6), indicating that any damage is confined to a thin surface layer; (ii) the strongest qualitative contrasts in crease evolution arise specifically in high-dimer content samples subjected to 254 nm during swelling (Figure 10), consistent with a cleavage-dependent response; and (iii) the strongest attenuation of 254 nm within ≈25 μm of the specimen surface (Figure S12 and Table S1) implies that the affected region is shallow relative to the 2 mm thickness. We therefore interpret cleavage-driven acceleration of surface swelling and stress relaxation as the dominant mechanisms while noting that small, UV-induced modifications to the near-surface network cannot be completely ruled out.

Together, these results (summarized in Table S3) demonstrate that postcure photomodulation, especially *in situ* 254 nm irradiation, provides an effective means of controlling surface creasing through dynamic manipulation of the network’s cross-linking state during swelling. The ability to tune crease morphology in real time by moving from dimerized to cleaved CoumAc cross-linking highlights the potential for spatiotemporal programming of surface patterns in photoresponsive polymer gels. Therefore, this PEGCoumAc gel is considered a worthwhile candidate for future investigations into engineered control of interfacial behavior.

## 4 Conclusions

This study demonstrates that coumarin-functionalized PEG gels provide a versatile platform for dynamically tuning mechanical properties, swelling behavior, and surface topography through controlled UV irradiation. By integrating permanent (PEGDA) and photoresponsive (CoumAc) cross-linkers within a single network, we modulate cross-link density postcure via light-driven coumarin dimerization and cleavage. Surface crease imaging revealed that CoumAc incorporation and photopolymerization conditions (wavelength, intensity, duration) strongly influence instability morphology, density, and evolution during swelling. These findings indicate that dynamic cross-links alter internal stress-relaxation pathways and can encode distinctive topographic patterns. Furthermore, irradiation of a 365 nm cured gel *in situ* with 254 nm UV light was shown to accelerate network relaxation and surface creases disappeared more quickly.

Swelling experiments show that CoumAc is a less efficient swelling-resistant cross-linker than PEGDA, resulting in higher equilibrium swelling ratios when PEGDA is replaced. Nevertheless, postcure 365 nm dimerization significantly increases stiffness, by up to 69%, on hour or subhour time scales, demonstrating that small CoumAc loadings can produce substantial, rapid mechanical reinforcement. Real-time FTIR confirmed that both dimerization and cleavage occur in the bulk network, though kinetics are sensitive to the irradiation protocol and initial network structure. Future work aims to further explore these spectroscopic data to quantify the extent of the dimerization and cleavage reactions.

Importantly, DMA cycling experiments revealed that bulk softening via 254 nm cleavage is minimal under current irradiation conditions, likely due to 254 nm attenuation through the sample, suggesting that practical property modulation is surface-confined for cleavage but bulk-accessible for dimerization. Accounting for UV attenuation in bulk samples is key to the future design of photoresponsive hydrogels, as attenuation can limit the depth and degree of cross-linker activation or cleavage, especially for wavelengths with high absorbance but low penetration (254 nm).

The dual-cross-linked PEG-coumarin system presented here is particularly well suited for applications requiring remote, reversible control over surface mechanics without loss of bulk integrity. Compared with approaches that rely on fillers, multiphase architectures, or noncovalent photoswitches to regulate performance, the coumarin-functionalized PEG gels studied here achieve large, reversible changes in stiffness and distinct surface patterns primarily through network-embedded, photoreversible covalent cross-links, providing a complementary route to photoprogrammed soft materials. For example, in smart hydrogel coatings, localized 365 nm irradiation could be used to stiffen and stabilize surface features under flow or contact, while targeted 254 nm exposure enables surface stress relaxation and smoothing. Similarly, in soft microactuators or valves, postcure dimerization provides a mechanism for mechanical locking after deployment, increasing force output or shape retention without additional material processing.

At the same time, several challenges must be addressed for practical implementation. Chief among these is the limited penetration depth of 254 nm light, which confines effective cleavage to near-surface regions and limits bulk softening. Long-term stability, potential photochemical fatigue, and the scalability of irradiation protocols also warrant further investigation. Strategies such as red-shifting coumarin derivatives, incorporating two-photon activation, or reducing the sample thickness may mitigate these limitations.

Despite these challenges, the strong coupling demonstrated here between photochemistry, bulk mechanics, and swelling-induced surface instabilities highlights the broader potential of coumarin-based dynamic cross-linking as a design tool for adaptive soft materials. By enabling spatiotemporal programming of the stiffness and surface topology, this platform opens new opportunities in responsive coatings, reconfigurable soft devices, and mechanically active biomaterials.

## Supplementary Material



## Notes

Reaction scheme for CoumAc synthesis; ^1^H NMR and COSY NMR spectra and mass spectrometry for CoumAc and intermediate product; experimental setup for *in situ* swelling microscopy; representative photopolymerization conversion and rate curves; original crease microscopy images without tracing; early time swelling ratio *Q*(*t*) data; 254 nm attenuation simulation and intensity measurements through gels; storage modulus at 26 °C for all formulations and cure conditions; summary of the impact of curing protocol on gel properties (PDF)
