# Supplementary material for: Photoswitchable Cross-Linking in Polymer Gels: Effects on Surface Creasing and Network Relaxation during Swelling
Source: Macromolecules. 2026 Mar 26;59(7):4622–35. doi: 10.1021/acs.macromol.5c03103 (PMC13085845; doi:10.1021/acs.macromol.5c03103)
Supplement: Supplementary file 1 [file ma5c03103_si_001.pdf]

Supplementary Information

Photo-Switchable Cross-Linking in Polymer Gels: Effects on Surface Creasing and Network Relaxation during Swelling

Alyssa VanZanten[a], Surbhi Punhani-Schillinger[a], M. Reed Blocksome[a], Aditya Ketkar[a], Shih-Yuan Chen[b], Michelle M. Driscoll[b], Robert C. Ferrier, Jr.[a], Caroline R. Szczepanski[a]*

[a]Department of Chemical Engineering & Materials Science, Michigan State University, East Lansing, MI 48824

[b]Department of Physics & Astronomy, Northwestern University, Evanston, IL, 60208

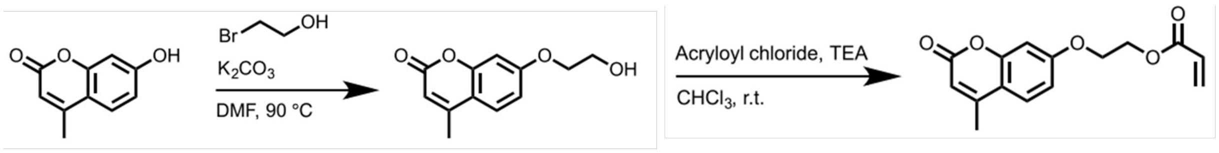

4-methylumbelliferone   7-(2-hydroxyethoxy)-4-methylcoumarin   7-(2-acryloyloxyethoxy)-4-methylcoumarin

Figure S1: Reaction scheme for the synthesis of 7,2-(acryloyloxyethoxy)-4-methylcoumarin.

^{1}H NMR (500 MHz, dmso) δ 7.69 – 7.63 (m, 1H), 6.98 – 6.92 (m, 2H), 6.18 (t, J = 1.2 Hz, 1H), 4.07 (t, J = 4.8 Hz, 2H), 3.72 (t, J = 4.8 Hz, 2H), 2.37 (d, J = 1.2 Hz, 3H).

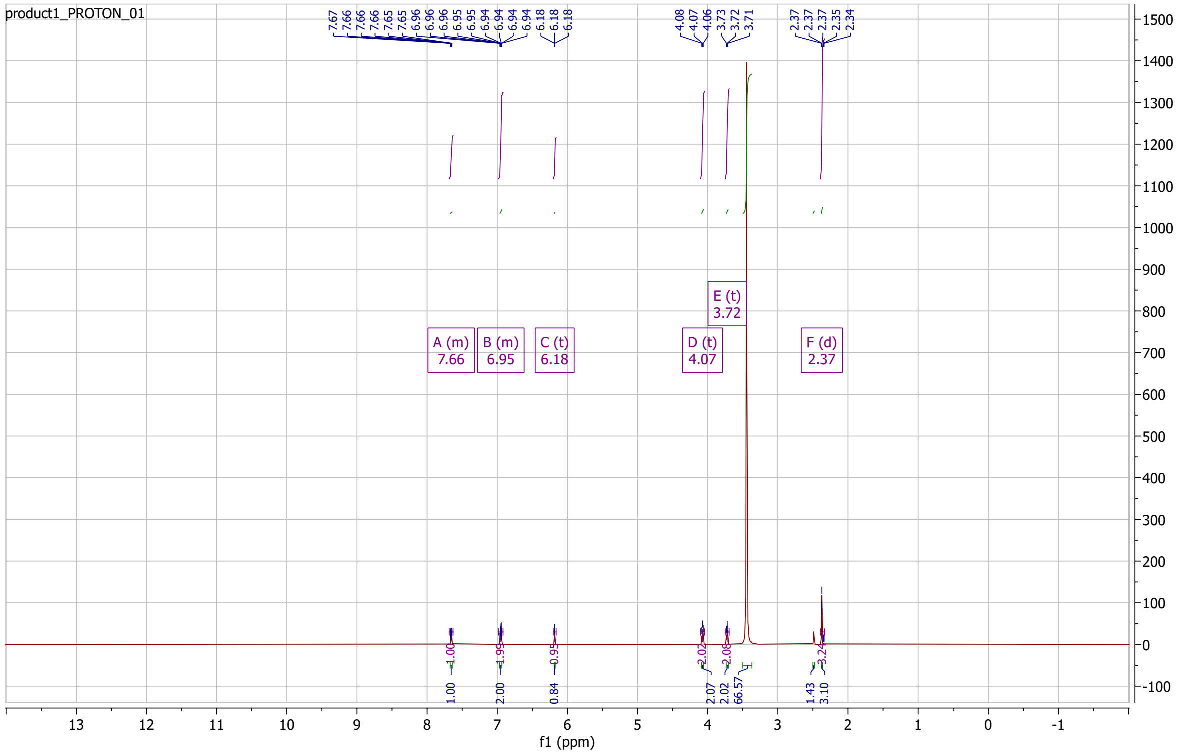

Figure S2: H-NMR spectrum of intermediate product (7-(2-hydroxyethoxy)-4-methylcoumarin) during CoumAc synthesis

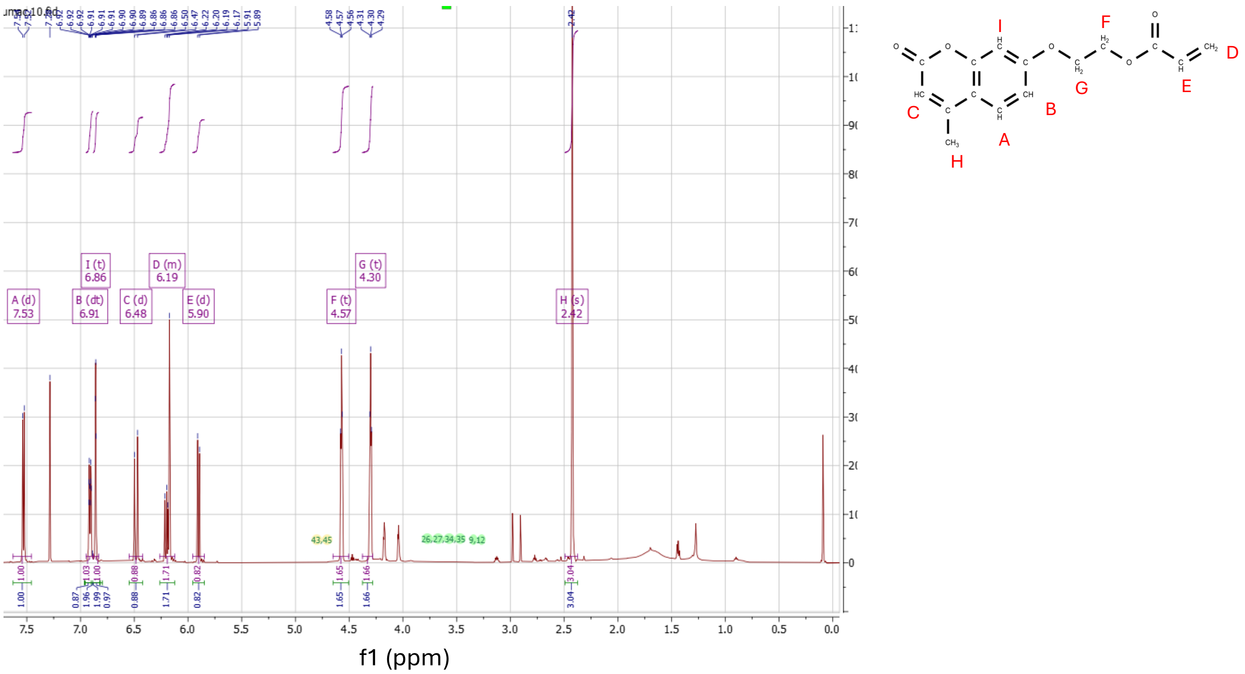

Figure S3: H-NMR spectrum of final CoumAc product (7-(2-acryloyloxyethoxy)-4-methylcoumarin).

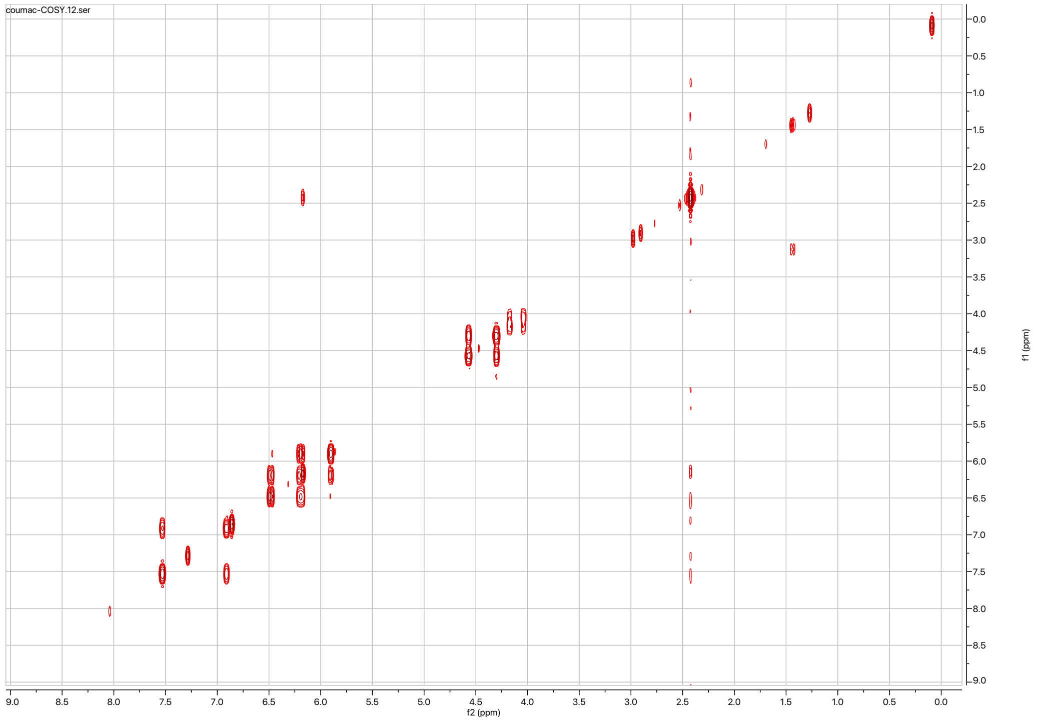

Figure S4: Full COSY NMR spectrum of the synthesized coumarin monomer - CoumAc. Structural correlations are highlighted in the analysis below.

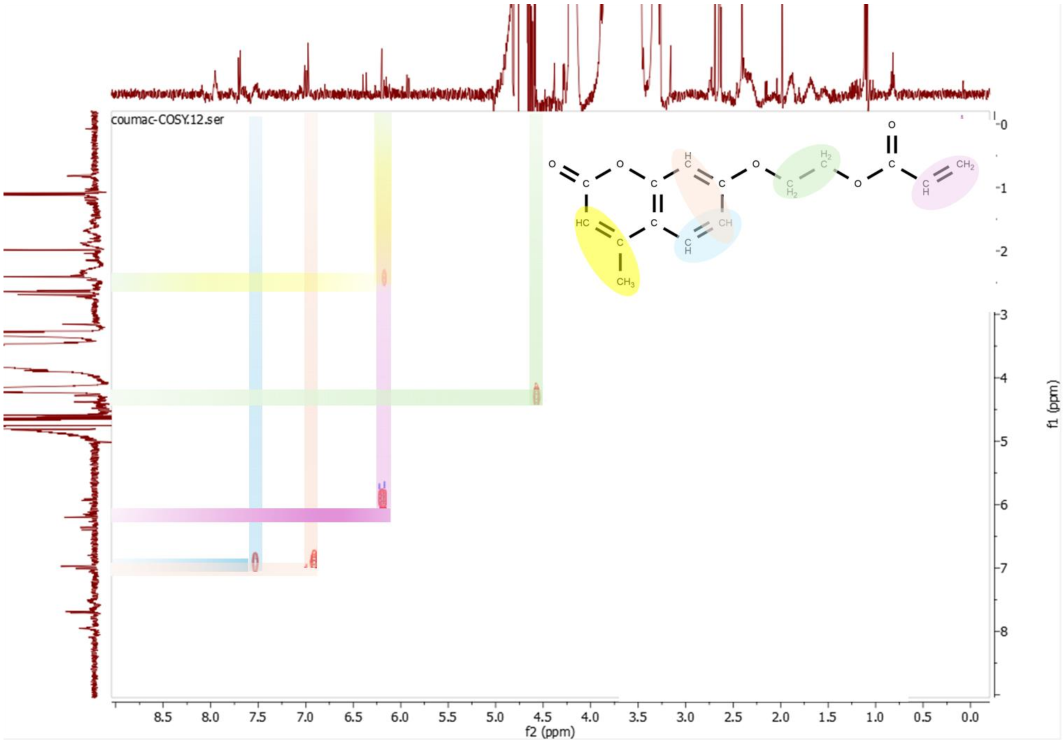

Figure S5: COSY NMR spectrum of the synthesized coumarin monomer - CoumAc. The centerline and mirrored correlations are removed to highlight major structural correlations. The full spectrum can be seen above. Here, the colored lines highlight the connections between the various functional groups highlighted with the corresponding color in the chemical structure schematic. The correlations are consistent with the anticipated structure of the synthesized coumarin monomer.

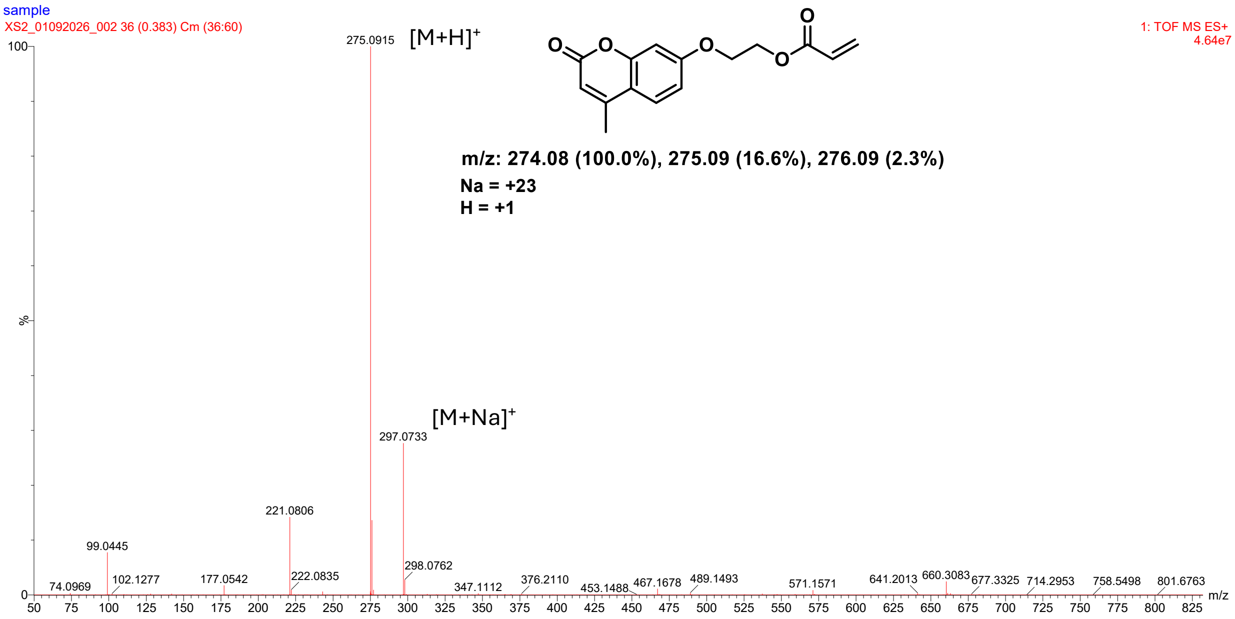

Figure S6: Resulting MS data from the synthesized coumarin monomer. The m/z is consistent with the predicted structure of the monomer plus H or Na ions. The chemical structure along with the predicted m/z is included in the plot.

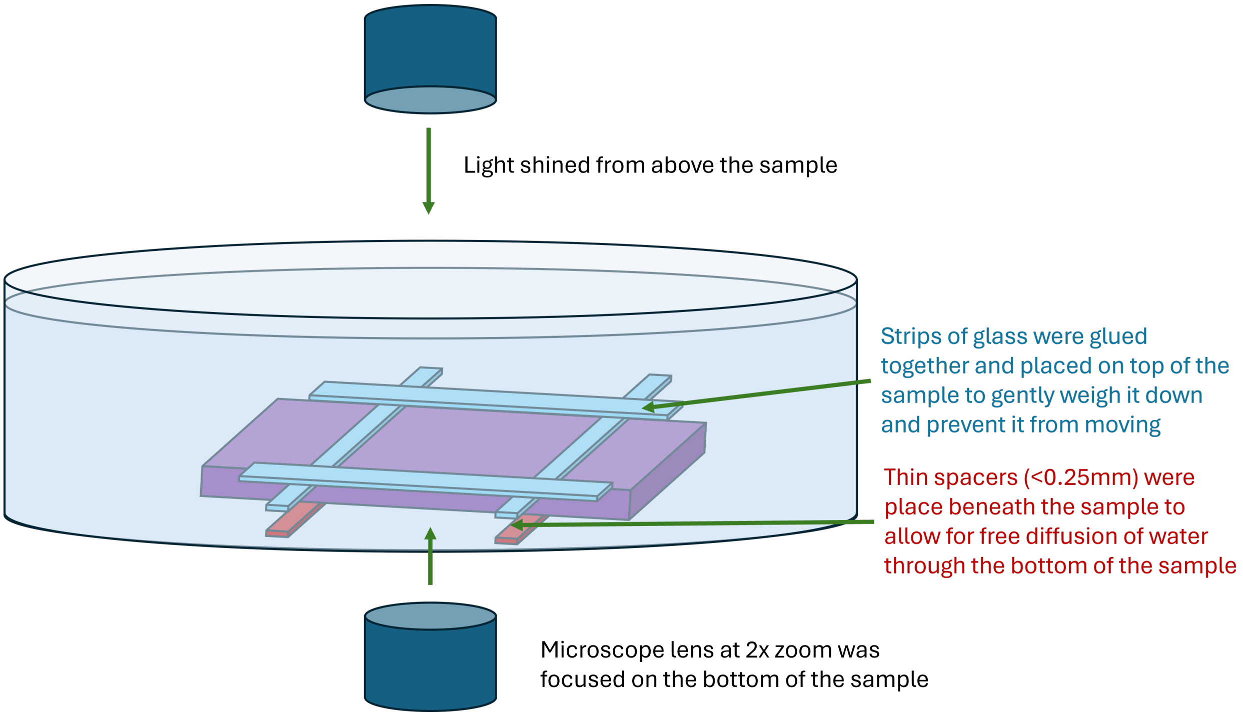

Figure S7: A description of the setup used to ensure swelling samples did migrate during microscope imaging.

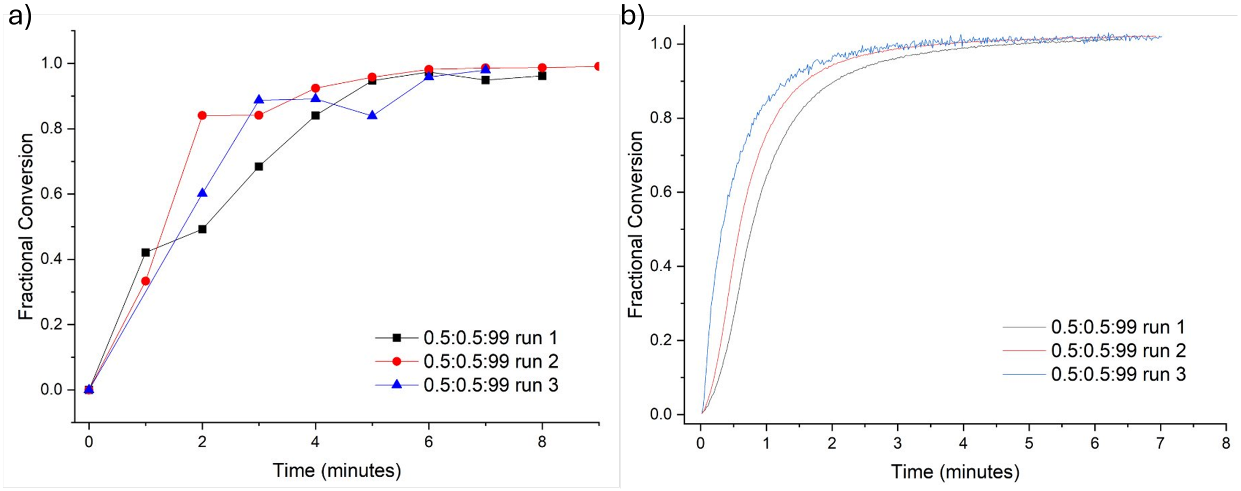

Figure S8: Examples of the polymerization curves collected during curing. a) The pseudo-real-time 254 nm and **b)** real-time 365 nm polymerization kinetics are compared for the 0.5:0.5:99 PEGCoumAc formulation.

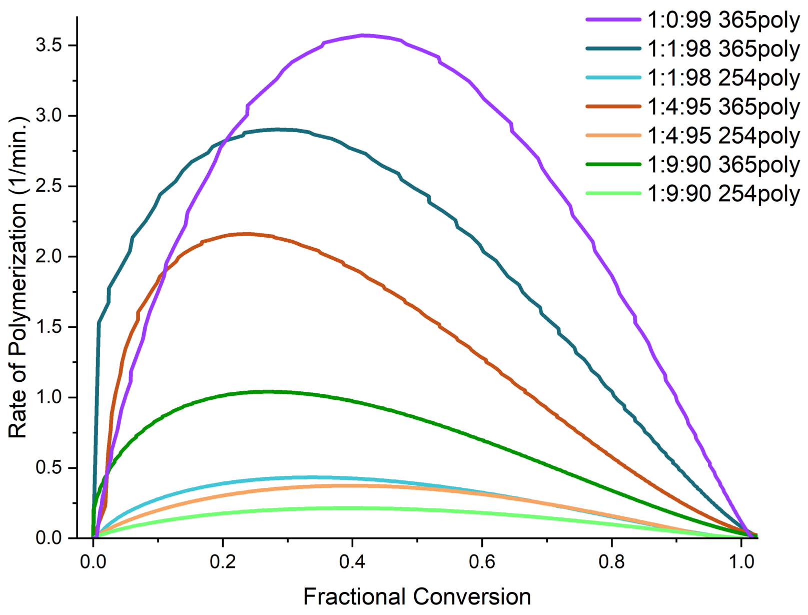

Figure S9: Rate of polymerization, calculated as the first derivative of fractional conversion versus time, is plotted versus fractional conversion for various PEGCoumAc formulation and polymerization conditions.

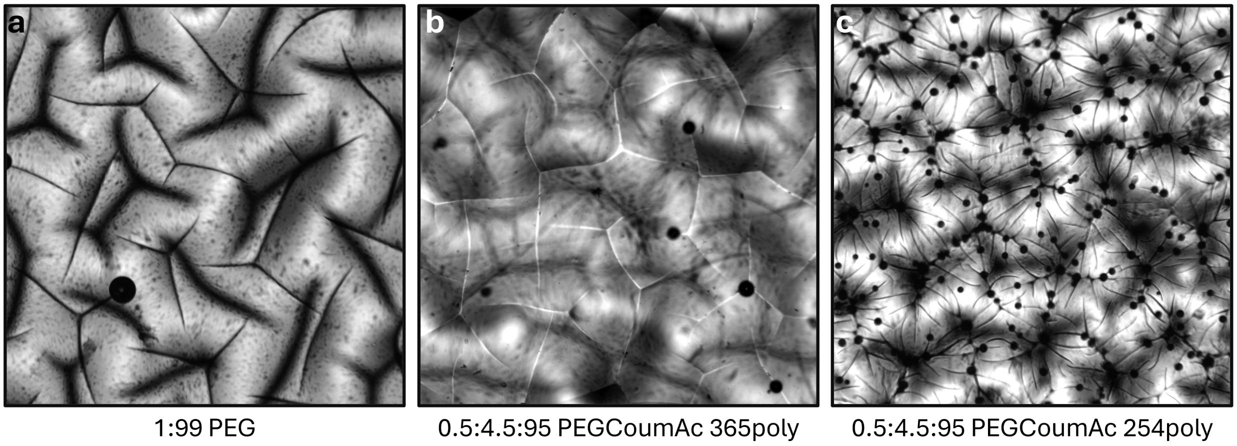

Figure S10: Original microscope images without tracing for **a)** a 1:99 PEG sample, **b)** a 0.5:4.5:95 PEGCoumAc sample polymerized at 365 nm, and **c)** a 0.5:4.5:95 PEGCoumAc sample polymerized at 254 nm. All images are at 320 seconds of swelling and show a field of view 3.328 x 3.328 mm.

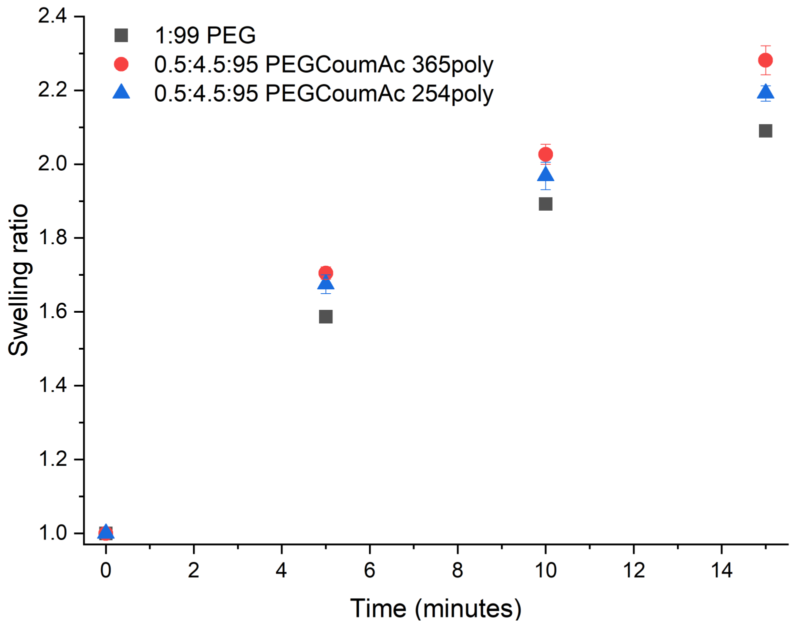

Figure S11: Swelling ratio (Q) during the first 15 minutes of swelling is included for the 1:99 PEG formulation as well as the 0.5:4.5:95 PEGCoumAc formulation under either 365 nm or 254 nm polymerization conditions.

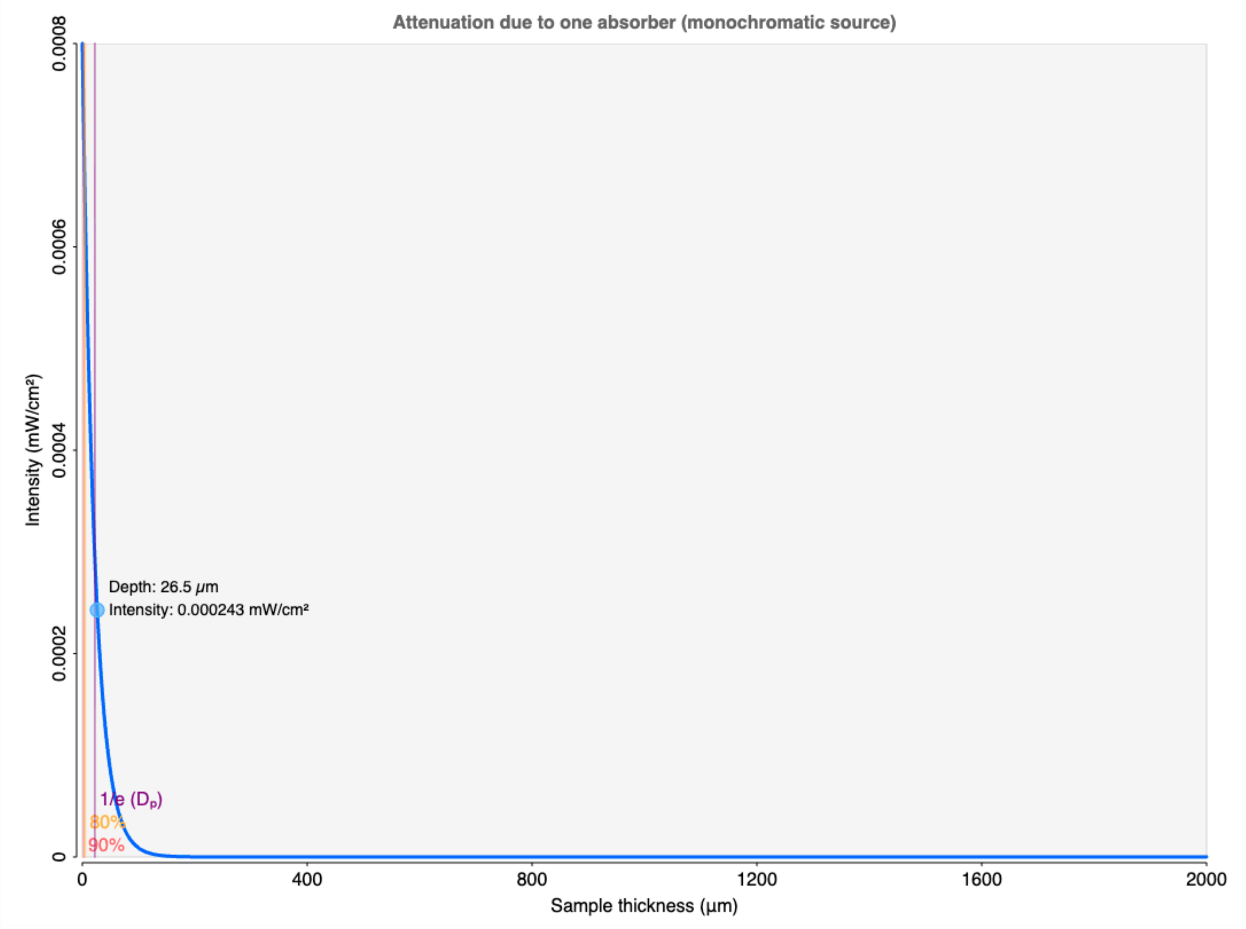

Figure S12: Attenuation of 254 nm light in a 2 mm thick specimen is estimated using an open-source tool [69] with inputs associated with our experimental protocol (i.e. incident intensity, initiator absorptivity, etc.). Notably, 254 nm irradiation intensity is decreased by 90% at a depth of $\sim 25\ \mu m$.

Table S1:

Intensity measurements after light passes through a glass slide, a PEGCoumAc sample, and a glass slide and PEGCoumAc sample together.

	254 nm UV oven intensity ($\frac{W}{cm^2}$)	365 nm UV lamp intensity ($\frac{W}{cm^2}$)
Raw measurement (no interference)	$8.5E10^{-3}$	0.1
Through glass	$1E10^{-4}$	0.09
Through 1:9:90 PEGCoumAc sample (2 mm)	$1.9E10^{-4}$	0.02
Through glass and sample	$1.5E10^{-4}$	0.02

Table S2: The storage modulus at 26°C values for all PEGCoumAc and some PEG formulations are included for both the 365 nm and 254 nm polymerization conditions.

Photo-polymerized at 365 nm		
PEGDA (mol%)	CoumAc (mol%)	Storage Modulus at 26°C (MPa)
0.5	0.5	0.044±0.0015
0.5	4.5	0.0677±0.0084
1	0	0.2252±0.194
1	1	0.0938±0.0039
1	4	0.1332±0.0129
1	9	0.1335±0.0039
5	0	0.9491±0.0986
5	5	1.0981±0.1203
5	15	0.9597±0.0109
10	0	1.803±0.0901
Photo-polymerized at 254 nm		
PEGDA (mol%)	CoumAc (mol%)	Storage Modulus at 26°C (MPa)
0.5	0.5	0.0868±0.0082
0.5	4.5	0.1039±0.0043
1	1	0.1625±0.0076
1	4	0.1748±0.0046
1	9	0.2805±0.1110
5	5	0.9625±0.0461
5	15	1.2753±0.0522

Table S3: Summary of results regarding expansion rate, crease morphology, and modulus.

	CoumAc Gel - Brief 365 nm Cure (7 min)	CoumAc Gel - Long 365 nm Cure (77 min)	CoumAc Gel - Brief 254 nm Cure (7 min)	CoumAc Gel - Long 365 nm Cure (77 min)
Post-Cure Protocol	None	None	None	254 nm (during swelling)
Swelling Behavior (Q_{eq}, $Q(t)$)	Higher Q_{eq} and slower $Q(t)$ compared to PEG control	Higher Q_{eq} and slower $Q(t)$ compared to PEG control	Lower Q_{eq} and similar $Q(t)$ compared to 365 nm counterpart	Higher Q_{eq} and slower $Q(t)$ compared to PEG control
Surface Morphology at 320 s swelling	Branched, modestly higher crease density than PEG control	Branched, modestly higher crease density and longer creases than PEG control	Brush-like, very high crease density, distinct morphology	Creases rapidly disappear with post-cure irradiation, absent by 185 s
Room Temperature Modulus (Trend)	Similar to PEG control	Young's modulus increases by 69% compared to brief cure due to dimer content	Higher modulus than 365 nm-cured analogue	n/a
